# Inferring tumor absolute copy number and clonal substructure from single-cell chromatin accessibility

**DOI:** 10.1093/bib/bbag265

**Published:** 2026-05-27

**Authors:** Ying Wang, Yuhao Deng, Hang Li, Xinbao Yin, Yanru Zhang, Yurong Chen, Min Zhang, Xin Wang, Zhizhuo Cao, Shaojun Zhang

**Affiliations:** Guangdong Academy of Medical Sciences and Medical Research Institute, Guangdong Provincial People’s Hospital (Guangdong Academy of Medical Sciences), Southern Medical University, No. 106 Zhongshan 2nd Road, Yuexiu District, Guangzhou 510080, China; Center for Endemic Disease Control, Chinese Center for Disease Control and Prevention, Harbin Medical University, No. 157 Baojian Road, Nangang District, Harbin 150081, China; Institute of Precision Medicine, The First Affiliated Hospital, Sun Yat-Sen University, No. 58 Zhongshan Er Road, Yuexiu District, Guangzhou 510080, China; Guangdong Academy of Medical Sciences and Medical Research Institute, Guangdong Provincial People’s Hospital (Guangdong Academy of Medical Sciences), Southern Medical University, No. 106 Zhongshan 2nd Road, Yuexiu District, Guangzhou 510080, China; Department of Urology, The Affiliated Hospital of Qingdao University, No.16 Jiangsu Road, Shinan District, Qingdao 266000, Shandong, China; Institute of Precision Medicine, The First Affiliated Hospital, Sun Yat-Sen University, No. 58 Zhongshan Er Road, Yuexiu District, Guangzhou 510080, China; Department of Gynecological Radiotherapy, Harbin Medical University Cancer Hospital, No. 150 Haping Road, Nangang District, Harbin 150081, China; Institute of Precision Medicine, The First Affiliated Hospital, Sun Yat-Sen University, No. 58 Zhongshan Er Road, Yuexiu District, Guangzhou 510080, China; Institute of Precision Medicine, The First Affiliated Hospital, Sun Yat-Sen University, No. 58 Zhongshan Er Road, Yuexiu District, Guangzhou 510080, China; Institute of Precision Medicine, The First Affiliated Hospital, Sun Yat-Sen University, No. 58 Zhongshan Er Road, Yuexiu District, Guangzhou 510080, China; Guangdong Academy of Medical Sciences and Medical Research Institute, Guangdong Provincial People’s Hospital (Guangdong Academy of Medical Sciences), Southern Medical University, No. 106 Zhongshan 2nd Road, Yuexiu District, Guangzhou 510080, China

**Keywords:** single-cell, copy number, clonal architectures, tumor heterogeneity, scATAC-seq

## Abstract

Accurate inference of absolute copy numbers beyond simple gains and losses from single-cell chromatin accessibility (scATAC-seq) data remains challenging, thereby obscuring the distinction between genetic and epigenetically driven oncogenic dependencies. Here, we present TeaCNV, a computational framework that reconstructs clonal absolute copy number profiles and tumor clonal architectures from scATAC-seq data without matched DNA baselines. Through validation both *in silico* and against bulk whole-genome sequencing in renal cell carcinomas, TeaCNV resolved subclonal absolute copy number profiles with less than 10% error and detected copy number variations (CNVs) with 98.6% accuracy, outperforming existing methods. Applied to six cancer types including renal, breast, pancreatic, head and neck, colorectal, and ovarian cancers, TeaCNV delineated polyclonal architectures and revealed distinct chromatin accessibility patterns driven by CNVs in key driver genes, including *AKT2*, *ZNF217*, and *SOX2.* By enabling absolute copy number profiling and clonal deconvolution from epigenomic assays, TeaCNV bridges critical gaps in studying oncogenic dependencies and genotype–phenotype relationships at single-cell resolution.

## Introduction

Chromosomal copy number variations (CNVs) are fundamental genomic alterations that drive tumor evolution by reshaping transcriptional landscapes [1, 2]. Aneuploidy, defined as the presence of abnormal chromosome numbers, is a hallmark of human cancer and is strongly associated with tumor aggressiveness and poor clinical outcomes [3–6]. Therefore, precise quantification of chromosomal absolute copy numbers (CNs) is crucial for understanding cancer evolution and informing clinical decision-making [7, 8].

Bulk and single-cell whole-genome sequencing (scWGS) remain the gold standards for CNV profiling [9–11], but both techniques have limitations. Bulk WGS enables quantification of tumor ploidy, yet offers limited resolution for clonal substructure deconvolution, particularly in detecting rare clones [8, 12]. Although scWGS improves clonal substructure resolution by providing absolute CN quantification at the single cell level, it cannot characterize the functional consequences of heterogeneous CNVs due to the absence of gene expression data [10, 13]. Moreover, both bulk WGS and scWGS fail to capture the dynamic interactions within the tumor microenvironment [14, 15], and face significant challenges in clinical implementation, including technical complexity, high cost, and limited scalability [16].

Single-cell RNA sequencing (scRNA-seq) has emerged as a powerful alternative, enabling integrated analysis of both microenvironment composition and tumor subpopulation architecture [17, 18]. Despite challenges such as transcriptional noise and posttranscriptional regulation [19], computational tools including inferCNV [20–24] and HoneyBADGER [25] have demonstrated the feasibility of detecting CNVs from scRNA-seq data at large genomic scales. Recent methodological advances, such as CopyKAT’s clonal substructure analysis [26] and allele-specific CNV detection by Numbat [27] and XClone [28], further enhance the ability to dissect tumor heterogeneity and link CNVs to their transcriptional consequences. However, scRNA-seq’s reliance on transcriptional readouts limits its ability to directly dissect the epigenomic contributions to tumor cell diversity.

Single cell ATAC sequencing (scATAC-seq), which profiles chromatin accessibility through transposase-accessible DNA sequencing, provides an opportunity to study malignant regulatory programs at single cell level [29, 30]. Characterizing epigenomic regulatory mechanisms in cancer requires careful consideration of CNV effects, as chromosomal alterations can substantially alter chromatin accessibility patterns in affected regions [29]. scATAC-seq offers unique advantages for CNV detection by directly measuring DNA accessibility rather than relying on indirect transcriptional outputs, thereby reducing susceptibility to gene regulatory confounders [31, 32]. Recently, computational methods like epiAneufinder [33] and Copy-scAT [34] have been developed to identify CNVs from scATAC-seq data. However, due to the extreme sparsity of scATAC-seq data which exceeds that of scRNA-seq data, and systematic biases at chromatin regulatory elements (CREs) such as promoters and enhancers, these methods are limited to identifying relative gain/loss at large genomic scales rather than absolute CN quantification [33–35]. This limitation hinders the understanding of clonal substructures and the ability to distinguish between the effects of genomic and epigenomic changes in polyploid tumors.

To address these limitations, we developed TeaCNV, a computational framework that quantifies absolute CN profiles and resolves clonal substructures directly from scATAC-seq data. Validated on both simulated datasets and bulk WGS, TeaCNV achieved 98.6% accuracy of CNVs calling and less than 10% error in absolute CN profiling, while detecting subclonal CNVs undetectable by bulk WGS. Application to multi-cancer types (renal, breast, pancreatic, head and neck, ovarian, and colorectal cancers) revealed lineage-defining amplifications of oncogenic drivers and decoupled genomic from epigenomic contributions within polyclonal architectures.

## Methods

### Human specimens

Four ccRCC patients were collected with informed consent approved by the Institutional Ethics Committee of the Affiliated Hospital of Qingdao University, including two patients for scATAC-seq, two for scATAC&RNA-seq co-assays, and three patients for bulk WGS.

### Experimental methods

#### Nuclei isolation, library preparation, and sequencing

Tissues were dissected, snap-frozen in liquid nitrogen and stored at −80°C. Nuclei were isolated from frozen tissues following the manufacturer’s protocols for scATAC-seq (10× Genomics, CG000212 Rev B) and scATAC&RNA-seq co-assays (10× Genomics, CG000375 Rev C), with RNase inhibitors in the buffer to prevent mRNA degradation during cell lysis.

Nuclei and barcoded beads were then loaded onto the 10× Genomics platform using the Chromium Next GEM Single Cell ATAC Library Kit v2 for scATAC-seq (CG000496 Rev A) and the Single Cell Multiome ATAC + Gene Expression kit for scATAC&RNA-seq co-assays (CG000338 Rev F), according to the manufacturer’s instructions. Barcoded libraries were pooled and sequenced with paired-end reads on Illumina NovaSeq 6000.

#### Library preparation and sequencing for bulk WGS

High-quality genomic DNA (~0.6 μg) was sheared with Covaris LE220 Sonicator (Covaris) to ~350 bp. The library was constructed using protocol of KAPA Hyper Prep kit (Roche). Fragmented DNA was purified using sample purification beads, and the product was repaired by the end and A base was added to the 3′ end. Then, the adapters are ligated with the specific barcode sequence. The CleanNGS magnetic beads (CleanNA) were used to screen out incomplete connections and self-connecting products. Sequencing libraries were formed by PCR amplification using universal primers complementary to the adaptor sequences. Paired-end sequencing was performed using the NovaSeq 6000 S4 Reagent Kit v1.5 (300 cycles) on Illumina NovaSeq 6000 platform (Illumina, San Diego, USA) by Sequanta Technologies (Shanghai, China).

#### scATAC-seq and scATAC&RNA-seq data processing

Reads from scATAC-seq and scATAC&RNA-seq were aligned to GRCh38 (hg38) and quantified using *cellranger-atac count* (v.1.2.0) and *cellranger-arc count* (v.2.0) pipelines (10× Genomics), respectively. Peaks were called using MACS3 (v.3.0.0) [36] through the *CallPeaks* function in Signac (v.1.14.0) [37]. Peaks on chromosomes X/Y and in the ENCODE Unified GRCh38 Blacklist were removed using “blacklist_hg38_unified” in the *subsetByOverlaps* function in Signac. The filtered, sample-specific peaks were used to construct peak-by-count matrices using *FeatureMatrix* in Signac for downstream analyses.

Quality control was performed using Signac. Cells were filtered based on the following criteria: number of fragments in peaks >1000, number of peaks in cell >2000, and enrichment-score for Tn5-integration events at transcriptional start sites >3.

#### Normalization, dimensionality reduction, clustering, and cell classification

The filtered peak-by-count matrices derived from scATAC-seq or scATAC&RNA-seq data were normalized using term frequency-inverse document frequency (TF-IDF) as implemented in Signac. The top 95% of peaks were selected as features for dimensionality reduction. Latent semantic indexing (LSI) was then performed using the RunSVD function, and LSI components 2–30 were used as input for UMAP embedding (RunUMAP, Seurat). Nuclei were subsequently clustered using a graph-based clustering approach in Seurat, also based on LSI components 2–30.

For scATAC-seq data, cell types were annotated based on gene activity scores of canonical lineage markers: immune cells were identified by high activity of *PTPRC*, endothelial cells by *PECAM1* and *CD34*, fibroblast cells by collagen genes (e.g. *COL1A1*, *COL1A2*, *COL3A1*), and epithelial cells by *EPCAM* and *KRT* family genes. For scATAC&RNA-seq data, gene-count matrices were processed in Seurat (v.4.4.0) [38] for scaling, normalization, and identification of highly variable genes using default parameters. The number of significant principal components (PCs) was determined from the ElbowPlot, and the top 2000 highly variable genes and the first 30 PCs were used for unsupervised clustering. Cell types were then assigned based on the expression of canonical lineage marker genes.

#### Bulk WGS data processing and CNV analysis

Sequencing reads from bulk tumor tissue and matched normal tissues were aligned to GRCh38 using Burrows–Wheeler Aligner (BWA v0.7.17) software [39], and resulting BAM files were sorted and indexed with SAMtools [40]. The “runVarbin” module of CopyKit (v0.1.2) [41] was then used to count reads in 220-kb genomic bins defined on the GRCh38 assembly, with GC correction applied. Consistent with our scATAC-seq preprocessing, we excluded bins on chromosomes X/Y, as well as ENCODE blacklist regions to avoid sex-specific and artifactual signals. The log ratio of tumor to normal was computed for each bin and used for CNV calling, segmentation, and the inference of absolute CNs. Briefly, we fitted a Gaussian kernel smoothed density to the distribution of segment ratios to identify the diploid peak centered at ratio = 1. The one-copy increment was defined as the distance between the diploid peak and the nearest adjacent peak. Absolute CNs were then obtained by scaling each segment ratio by this increment and rounding to the nearest integer.

### TeaCNV algorithm

#### Preprocessing and transformation of scATAC-seq data

To balance sparsity and genomic coverage, we removed peaks detected in <5% of cells. If <10 000 peaks remained, we progressively relaxed this threshold until at least 10 000 peaks were retained. In solid tumors, non-epithelial cells (immune, endothelial, and fibroblast populations) were treated as non-malignant reference cells, while epithelial cells were treated as candidate malignant cells and used as inferred cells for CNV detection. For each group (reference and inferred), we retained cells whose total read counts and numbers of detected peaks fell between the 5th and 95th percentiles. Cells were further excluded if >60% of peaks on any chromosome had zero counts to reduce bias from chromosomal dropout.

We constructed two peak-by-cell matrices: ${X}_{n\times m}$ for reference cells and ${Y}_{n\times t}$ for inferred cells, where $n$ denotes the number of peaks ($n\ge 10\;000$), $m$ represents the number of reference cells, and $t$ is the number of inferred cells. Values in both matrices were truncated to the range [0,4]. To correct for sequencing-depth variation, we normalized each cell by its total counts and rescaled to the mean library size within each matrix, yielding ${\mathrm{X}}^{\prime }$ and ${\mathrm{Y}}^{\prime }$. For each peak *i*, we calculated the reference mean ${\overline{X\prime}}_{i\cdotp }$, and defined the ratio matrix as follows:


$$ {R}_{i,j}=\frac{Y{\prime}_{i,j}}{{\overline{X\prime}}_{i\cdotp }}\ \left(1\le i\le n,1\le j\le t\right), $$

which encoded relative accessibility in inferred cells compared with reference cells.

#### Aggregating cell subpopulations

To capture broad genomic patterns, we sorted peaks by genomic coordinate and, on each chromosome, averaged ratios within windows of five consecutive peaks (user-adjustable), obtaining a genomic-window matrix. Using Seurat, we identified top 2000 highly variable features, performed PCA and applied unsupervised clustering (resolution = 1) on the first 50 PCs yielded an initial set of inferred cell subpopulations (subgroups). For each subgroup *s*, we computed subgroup-level ratio for peak *i*:


$$ {\overline{R}}_{i\cdotp}^s= average\left[{R}_{i, k\epsilon S}\right] $$

#### Genome segmentation of cell subpopulations

For each subgroup *s*, we ordered ${R}^s$ by genomic coordinate and performed changepoint detection to identify chromosomal segments with homogeneous ratios. We primarily used the pruned exact linear time (PELT) algorithm in *changepoint* R package [42], with FPOP algorithm in *robseg* R package [43] as an alternative. Segments shorter than 2 Mb were excluded. For each retained segment $j$, the segmental ratio was defined as follows:


$$ {R}_j^s= median\left({\overline{R}}_{j_{k\in{segment}_j},\bullet}^s\right), $$

which served as the relative copy ratio for subgroup $s$ at segment $\,j$.

#### Inferring absolute copy number

For each subgroup, we obtained segmental ratios $R=\!\left\{{R}_j|\ j\in \left\{1,\dots, l\right\}\right\}$ over $l$ segments and modeled ${R}_j$ by a mixture distribution of integer CN states $Q=\left\{1,2,\dots, I\right\}$:


$$ P\left({R}_j|\mu, {\sigma}^2,\theta, {w}_j\right)={\sum}_{q\in Q}P\left(q|{\theta}_q,{w}_j\right)\bullet N\left({R}_j|{\mu}_q,{\sigma}^2\right) $$

where ${\mu}_q$ is the expected relative copy ratio for state $q$, ${\sigma}^2$ is a shared variance, ${w}_j$ is the genomic fraction of segment $j$, and $P\left(q|{\theta}_q,{w}_j\right)$ is the prior probability parameterized by ${\theta}_q$  $\left(q\in Q\right)$ and ${w}_j$. Following the maximum entropy principle, as the statistical hypothesis employed in ABSOLUTE [8], we set:


$$ P\left(q|{\theta}_q,{w}_j\right)=\frac{e^{-{\theta}_q\bullet{q}^{\#}\bullet{w}_j}}{\sum_{k\in Q}{e}^{-{\theta}_k\bullet{k}^{\#}\bullet{w}_j}} $$

where ${q}^{\#}$ and ${k}^{\#}$ respectively indicate the order of integer CN states $q$ and $k$ in $Q,$ starting from 1 (e.g. $q$ = 1 corresponds to ${q}^{\#}=1$). The expected genomic fraction of state $q$ across the genome is $\sum_{j=1}^l{w}_j\bullet P\left({q}_j|{\theta}_q,{w}_j\right).$

The unknown parameters $\theta =\left\{{\theta}_{q\in Q}\right\}$ were estimated by minimizing the following loss function using the Nelder–Mead optimization algorithm:


$$ \underset{\theta }{\hat{\theta}=\mathit{\arg}\min }{\left\{\sum_{q\in Q}{\left(\sum_{j=1}^l{w}_j\bullet P\left(q|{\theta}_q,{w}_j\right)-{\theta}_q\right)}^2\right\}}^{\frac{1}{2}} $$

With $\hat{\theta}$, we computed the prior probabilities $\hat{P}\left(q|{\hat{\theta}}_q,{w}_j\right)$.

Next, we estimated the expected relative copy ratio $\mu =\left\{{\mu}_q|q\in Q\right\}$ using maximum likelihood estimation. The full likelihood of relative copy ratio $R$ across the genome is:


$$ P\left(R|\mu, \sigma, \hat{\theta},w\right)=\prod_{j=1}^lP\left({R}_j|\mu, {\sigma}^2,\hat{\theta},{w}_j\right) $$

and the corresponding log-likelihood is:


$$ logL\left(R|\mu, \sigma, \hat{\theta},w\right)=\sum_{j=1}^l logL\left({R}_j|\mu, {\sigma}^2,\hat{\theta},{w}_j\right) $$

where


$$ L\left({R}_j|\mu, {\sigma}^2,\hat{\theta},{w}_j\right)={\sum}_{q\in Q}P\left(q|{\hat{\theta}}_q,{w}_j\right)\bullet N\left({R}_j|{\mu}_q,{\sigma}^2\right) $$

For segment $j$, the posterior probability of integer CN state $q\in Q$ is computed as:


$$ P\left( CN=q|{segment}_j\right)=p\left(q|{\hat{\theta}}_q,{w}_j\right)\bullet \frac{N\left({R}_j|{\hat{\mu}}_q,{\sigma}^2\right)}{\sum_{k\in Q}P\left(k|{\hat{\theta}}_k,{w}_j\right)\bullet N\left({R}_j|{\hat{\mu}}_k,{\sigma}^2\right)} $$

The final absolute CN state for segment $j$is defined as the CN state $q$ with the highest posterior probability.

To optimize absolute CN estimation, we computed differences between adjacent integer CN states:


$$ {\Delta}_q={\mu}_q-{\mu}_{q-1}\ \left(q=2,\dots, I\right), $$

which is theoretically constant and <1 (e.g. ${\Delta}_q\approx 0.5$ corresponds to a change of 1-copy relative to diploid). However, biases in ${\mu}_q$ estimation (e.g. due to uneven sequencing coverage or technique noise) may result in non-constant ${\Delta}_q$values across $q$ (Supplementary Fig. 17a).

To address this, we constructed a small set of candidate spacings $\left\{{\Delta }_i\right\}$, enforced a linear relationship for the expected copy ratio:


$$ {\mu}^{(i)}=\left\{{\mu}_q^{(i)}={\mu}_1+\left(q-1\right)\bullet{\Delta }_i|q\in \mathrm{Q}\right\} $$

**Figure 1:**
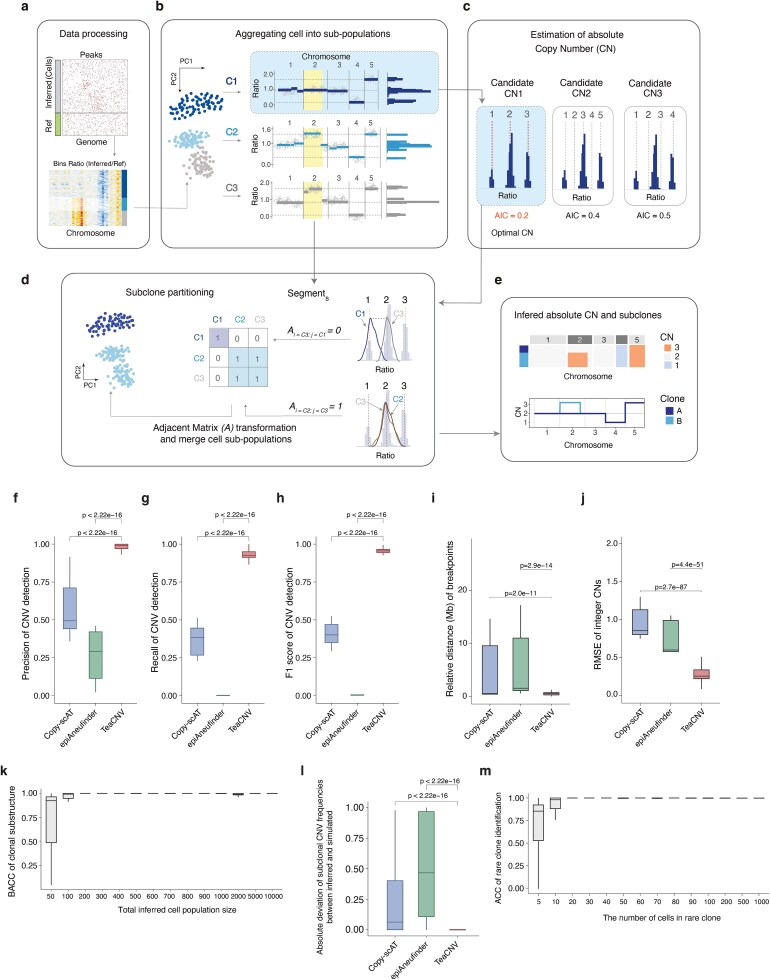
Overview of the TeaCNV algorithm and performance evaluation. (a) Workflow for preprocessing scATAC-seq data. TeaCNV converts the cell-peak matrix into copy ratio values relative to reference cells (Ref) and merges the ratios of adjacent peaks. (b) Cell clustering and genome-wide segmentation. For each cluster, the mean copy ratio across cells is calculated and subjected to PELT segmentation. The average copy ratio of bins within each segment is used to generate a histogram of genomic segments, in which bar length represents the genomic fraction (*x*-axis) of each segment. (c) Estimation of absolute CN. Three candidate interpretations of the copy ratio histogram (corresponding to cluster C1 in b) are shown in terms of absolute CNs. Dotted lines indicate copy ratios corresponding to specific absolute CNs. The optimal absolute CN estimation is determined according to the minimum AIC value. (d) Optimization of subclone partitioning. Clusters are merged when no significant differences in either absolute or relative CNs are detected across all genomic segments. (e) Representative TeaCNV output displaying subclonal absolute CN profiles. (f–h) Performance of CNV detection quantified by precision (f), recall (g), and F1 score (h) on simulated datasets. (i) Relative distance between inferred chromosomal breakpoints and the ground truth. (j) RMSE between inferred integer CNs and the ground truth. (k) BACC of subclone identification across datasets with varying population sizes. (l) Absolute deviation between inferred and simulated subclonal CNV frequencies. (m) Classification accuracy (ACC) for rare clone detection in simulated datasets.

The final absolute CN configuration for each subgroup was chosen as the candidate ${\mu}^{(i)}$ with the minimum Akaike information criterion (AIC) (Fig. 1c). Further details are provided in Supplementary Methods.

#### Tumor subclone partitioning and scoring

To avoid potential over-clustering, we constructed an adjacency matrix $A$ over subgroups. We initialized ${A}_{i,j}=1$ if $i=j$ and ${A}_{i,j}=0$ otherwise. For each subgroup ${s}_i$, we computed its mean normalized peak profile ${Y}_{s_i}^{\prime }$; for each pair $\left({s}_i,{s}_j\right)$, we formed the peak signal ratio:


$$ {R}_{s_i,{s}_j}=\frac{Y_{s_i}^{\prime }}{Y_{s_j}^{\prime }} $$

We segmented this profile and compared peak signals in each segment using two-sided t-tests with Benjamini–Hochberg adjustment. If any segment had adjusted $P$-value <.05 and differed in inferred CN between ${s}_i$ and ${s}_j$, we set ${A}_{i,j}={A}_{j,i}=0$. Otherwise, we updated ${A}_{i,j}={A}_{j,i}=1$. The final subclonal partitioning was derived from the updated adjacency matrix. For each subclone, we re-estimated integer CN states and computed ploidy as a length-weighted average:


$$ ploidy=\sum_{j=1}^l{w}_j\bullet{\hat{q}}_j $$

We evaluated the reliability of integer CN profiles by assuming that, in homogeneous subclones, observed copy ratios follow expected integer-state ratios (Supplementary Fig. 17b) and summarizing their mean squared error (MSE) from the expected values together with the genome fraction well explained by the model into a single composite score. This score additionally penalizes unrealistically small single-copy spacing, excessive segmentation and extreme ploidy (details in Supplementary Methods), such that higher score indicate more homogeneous and reliable subclone CN profiles.

### Application of other CNV inference approaches

For comparison, we applied epiAneufinder (v1.1.3) [33] and Copy-scAT (v0.4.0) [34] to scATAC-seq data from three ccRCC samples with matched bulk WGS, using default parameters at single-cell resolution. In addition, for two ccRCC samples with scATAC&RNA-seq, we employed inferCNV (v1.22.0) [20], Numbat (v1.3.2.1) [27], XClone (v0.3.8) [28], CopyKAT (v1.1.0) [26], and sciCNV [44] on the scRNA-seq data, using the same reference cells as in TeaCNV. inferCNV was run with recommended 10× settings (denoise = TRUE, cutoff = 0.1) and we additionally performed “consensus” i6 HMM mode (inferCNV-HMM) to infer integer CN profiles. For Numbat, SNP pileup data were generated following the official documentation, and Numbat was then run with default parameters on allele counts and gene expressions. XClone was run in default mode, combining its RDR and BAF modules to derive CNV profile estimates.

### Evaluating performance of copy number estimation

We evaluated CNV detection using all CNVs derived from bulk WGS as ground truth, regardless of event size. The genome was partitioned into non-overlapping 100-kb bins, and bins overlapping breakpoints in either the ground truth or inferred profiles were excluded. For each method, bins were classified as true positives (TP), false positives (FP), false negatives (FN), or true negatives (TN) according to the presence or absence of CNVs in the ground truth and inferred results. Precision, recall, accuracy, and F1 score were then computed using their standard definitions (see Supplementary Methods).

To assess reconstruction of polyclonal substructure, we calculated the balanced accuracy (BACC) [45] for each subclone based on TP, FP, TN, and FN, and summarized the overall performance as weighted average across subclones. Because TeaCNV reports CNVs at clonal level, precision, recall, and F1 score were averaged across the subclones within each sample. For epiAneufinder and Copy-scAT, CNV events were considered detected at the bulk level if present in at least 10%–100% of cells (10% steps), and evaluation metrics were averaged across thresholds.

We further quantified the accuracy of inferred CN profiles using the root mean square error (RMSE) between inferred and WGS-based integer CNs at the bin level. TeaCNV directly provided integer CNs, whereas epiAneufinder and Copy-scAT do not report integer CNs directly, we centered the inferred CNV scores to align with integer CNs for bins with distinct absolute CN states in WGS data. The definition of ${CN}_i$ was as follow:


\begin{align*} {CN}_i=\left\{\begin{array}{c}{CN}_i\kern0.75em for\ TeaCNV\\{}\!\!\!\!\! CNV\ {score}_i{-}\underset{j\in \left\{j|{CN}_j^{true}={CN}_i^{true}\right\}}{\mathrm{average}}\left( CNV\ {score}_j\right)+{CN}_i^{true}\kern0.75em\!\!\!\! for\ other\ methods\end{array}\right. \end{align*}

This adjustment allows for a comparison of inferred profiles against the ground truth.

Finally, to quantify how well distinct integer CN states can be distinguished, we proposed a dispersion score to measure the separation between inferred signal distributions corresponding to different integer CN states, normalized to $\left[0,1\right]$ (Supplementary Fig. 17c). Higher dispersion score indicates a better ability to distinguish distinct absolute CN states, reflecting a more accurate estimation of CN states (details in Supplementary Methods).

### Chromatin accessibility and downstream analysis

We analyzed differential chromatin accessibility in PDAC, BRCA, HNSCC, OV, and CRC samples and classified subclone-specific peaks as CNV-driven or CRE-regulated based on overlap with subclonal CNVs. Genes linked to clonal peaks were subjected to hallmark-based gene set from MSigDB [46] enrichment analysis to characterize subclone-specific pathways. Details are provided in Supplementary Methods.

## Results

### Methodology to infer clonal absolute copy number profile from scATAC-seq

We developed TeaCNV, a quantitative framework for reconstructing clonal absolute CN profiles and clonal substructure from scATAC-seq data without requiring matched DNA sequencing references. The algorithm processes an input peak-by-cell count matrix with cells pre-annotated as reference (normal) or inferred (tumor or epithelial) cells. Peaks are sorted by chromosomal coordinates. The outputs include clonal partitions of single cells and corresponding subclone-level absolute CN states for chromosomal segments (Fig. 1).

The inference pipeline operates through four steps (Methods). First is initial cell clustering. For each inferred cell, relative copy ratios are computed by normalizing peak accessibility signals against the median accessibility in matched reference cells. Dimension reduction via principal component analysis (PCA) on these ratios at the genomic window level enables initial clustering of inferred cells into subpopulations (Fig. 1a). Second is chromosomal breakpoint detection. Cells within each subpopulation are aggregated. Multi-scale chromosome breakpoints are identified using the PELT algorithm [47]. This generates segmentation boundaries for each subpopulation while accommodating scATAC-seq data sparsity (Fig. 1b). Third is joint ploidy-state optimization. For each subpopulation, absolute CNs of genomic segments are inferred through a combined optimization, including Nelder–Mead optimization to model expected relative copy ratios and CN state relationships, maximum likelihood estimation weighted by segment length and ratio variance, optimal integer CN determination via minimum AIC (Fig. 1c). Fourth is clonal architecture refinement. Subpopulations undergo pairwise comparison of their absolute and relative CN profiles. Subpopulations with concordant profiles are merged, followed by breakpoint recalculation and integer CN estimation (Fig. 1d). Last is the consensus profile generation, including clonal absolute CN profiles across chromosomal segments and single-cell clonal assignments (Fig. 1e).

### *In silico* evaluation

To evaluate the performance of TeaCNV, we simulated scATAC-seq data with well-defined ground truth using the simATAC [48] tool (Supplementary Methods). We created a series of simulated datasets with varying inferred cell population sizes (50 to 10 000 cells), clonal substructure complexity (1–5 subclones with prevalence from 10% to 90%), and CNV genomic scales (10% to 60%). Across all conditions and replicates, this yielded 680 simulated datasets (Supplementary Table S1). We randomly selected the subclonal CNV events, and a subclone with 10% frequency was designed as a rare clone. Besides TeaCNV, we also inferred CNV profiles using Copy-scAT [34] and epiAneufinder [33] (Supplementary Methods). The computational efficiency of the TeaCNV algorithm scales linearly with the number of cells (Supplementary Fig. 1a and b).

We compared CNV detection performance using precision, recall, and F1 score (Supplementary Methods; Supplementary Table S2). TeaCNV achieved significantly better CNV detection performance than the other methods (Fig. 1f–h), and its performance remained robust across CNVs of different genomic scales (Supplementary Fig. 1c–e). Additionally, TeaCNV accurately detected CNV boundaries, exhibiting the lowest deviation (mean: 0.57 Mb) and variance, although breakpoint deviation increased with the genomic fraction of CNVs (Fig. 1i and Supplementary Fig. 1f), with detected minimum CNV lengths spanning 2.04 to 4.20 Mb (Supplementary Fig. 1g). Together, TeaCNV achieved an average sensitivity of 0.96 for broad CNVs, including arm- and subarm-level events (Supplementary Fig. 2a). For focal CNVs, sensitivity declined as focal CNV size decreased, with an average sensitivity of 0.47 overall (Supplementary Fig. 2b).

We further calculated the RMSE of inferred integer CNs relative to the ground truth to measure the error of absolute CN quantification. TeaCNV showed the lowest error in integer CN quantification, with the maximum error no exceeding 0.5 (Fig. 1j and Supplementary Fig. 1h). We then evaluated the clonal substructure deconvolution performance across different population sizes using BACC across intra-tumor subclones (Supplementary Methods). TeaCNV accurately and robustly reconstructed clonal substructure with ≥200 cells (BACC >0.95, Fig. 1k). Moreover, the prevalence of subclonal CNVs inferred by TeaCNV was closer to the theoretical values (Fig. 1l and Supplementary Fig. 1i). Finally, we evaluated the sensitivity of rare clone detection by TeaCNV and found that at least 20 cells per subclone are required for robust rare clone detection (Fig. 1m and Supplementary Fig. 1j–l).

### Performance evaluation of TeaCNV using matched bulk WGS data in clear cell renal cell carcinoma

**Figure 2:**
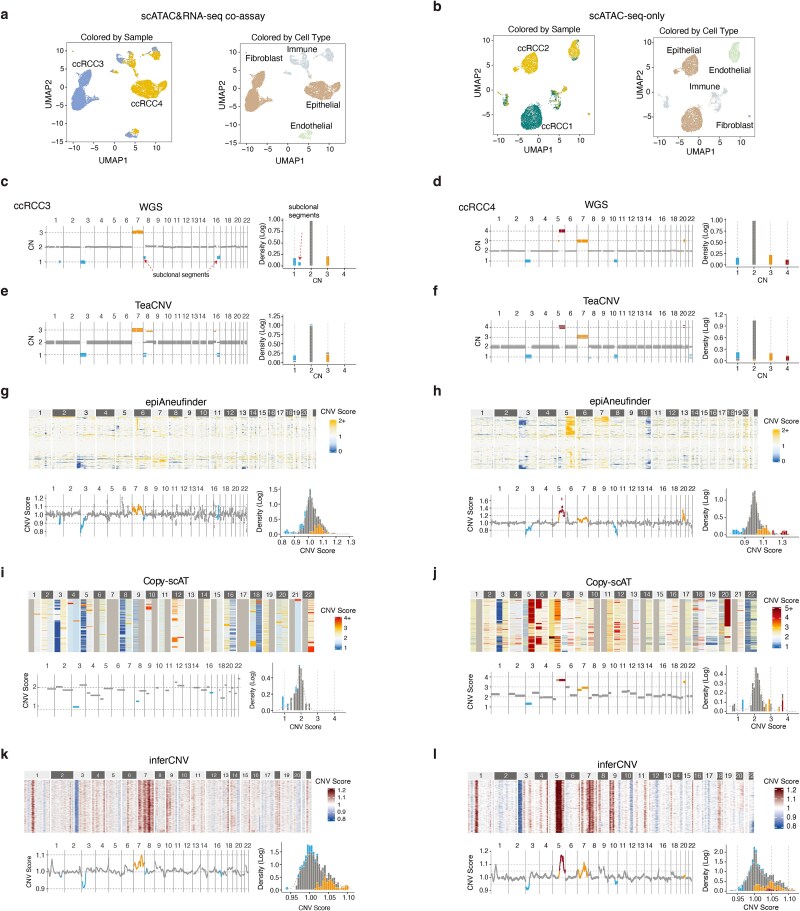
Comparison of the estimated copy number profiles with bulk WGS data. (a, b) UMAP plots of cells from ccRCC patients with (a) scATAC&RNA-seq co-assay data and (b) scATAC-seq-only data, colored by sample and cell type. (c, d) Integer CN profiles from bulk WGS data (left) and corresponding genome-wide distributions of absolute CN states (right) for ccRCC3 (c) and ccRCC4 (d). Significantly altered regions are highlighted in blue (loss, CN<2) and orange/red (gain, CN>2). (e, f) Clonal integer CN profiles estimated by TeaCNV (left) and genome-wide distributions of estimated integer CN states (right) for ccRCC3 (e) and ccRCC4 (f). In the left panel of each subfigure, bars of each genomic segment correspond to different clones. CNV segments are highlighted in blue (loss, CN<2) and orange/red (gain, CN>2). (g, h) Heatmaps of relative CN profiles (top) and genome distributions of relative CNV scores (bottom) estimated by epiAneufinder for ccRCC3 (g) and ccRCC4 (h). In the genome distribution plots, blue and orange/red highlights indicate CNV regions identified by bulk WGS data. (i, j) Copy-scAT estimates for ccRCC3 (i) and ccRCC4 (j). (k, l) inferCNV estimates for ccRCC3 (k) and ccRCC4 (l).

To evaluate TeaCNV’s ability to reconstruct clonal architecture of CNVs, we performed high-throughput 10× Genomics sequencing on four clear cell renal cell carcinoma (ccRCC) patients. This generated two scATAC&RNA-seq co-assay datasets and two scATAC-seq-only datasets (Fig. 2a and b, Supplementary Fig. 3). Cell type annotation was based on chromatin accessibility signatures of canonical markers (*PTPRC*, *PECAM1*, *CD34*, *COL1A2*, and *EPCAM*), followed by UMAP visualization, which identified 8367 epithelial cells for CNV analysis (Supplementary Figs 4 and 5).

Using non-epithelial cells (immune, endothelial, and fibroblast cells) as references, we applied TeaCNV to infer genome-wide clonal absolute CNs. To validate the performance of TeaCNV, we analyzed three ccRCC patients (ccRCC3, ccRCC4, and ccRCC1) with matched bulk WGS data. For the two scATAC&RNA-seq co-assay samples, the absolute CN profiles from bulk WGS data were considered the ground truth (Fig. 2c and d). The clonal integer CN profiles estimated by TeaCNV showed high concordance with WGS profiles (Fig. 2e and f). In ccRCC3, TeaCNV accurately identified the trunk events (chr3p loss and chr7 gain) and subclonal losses (chr8p and chr16q), which showed absolute CNs between 1 and 2 in bulk WGS data (Fig. 2c and e). Similarly, in ccRCC4, TeaCNV recalled all driver CNVs observed in bulk WGS data, including losses of chr3p/chr10q and gains of chr5q/chr7/chr22q (Fig. 2d and f).

For the scATAC&RNA-seq co-assay samples, we applied epiAneufinder and Copy-scAT to chromatin accessibility data, and inferCNV [20], Numbat [27], XClone [28], CopyKAT [26], and sciCNV [44] to the gene expression data (Fig. 2g–l, Supplementary Fig. 6). The scATAC-based approaches either diluted signals from aberrant regions or misidentified neutral regions, leading to ambiguous distributions of inferred CNV scores for segments with distinct CN states (Fig. 2g–j). The scRNA-based approaches exhibited differential biases: inferCNV recalled driver CNVs but also produced false positive signals in focal regions (Fig. 2k and l, Supplementary Fig. 6a), as did CopyKAT and sciCNV (Supplementary Fig. 6d and e), while Numbat and XClone missed positive signals due to their dependence on limited allelic information (Supplementary Fig. 6b and c**).**

**Figure 3:**
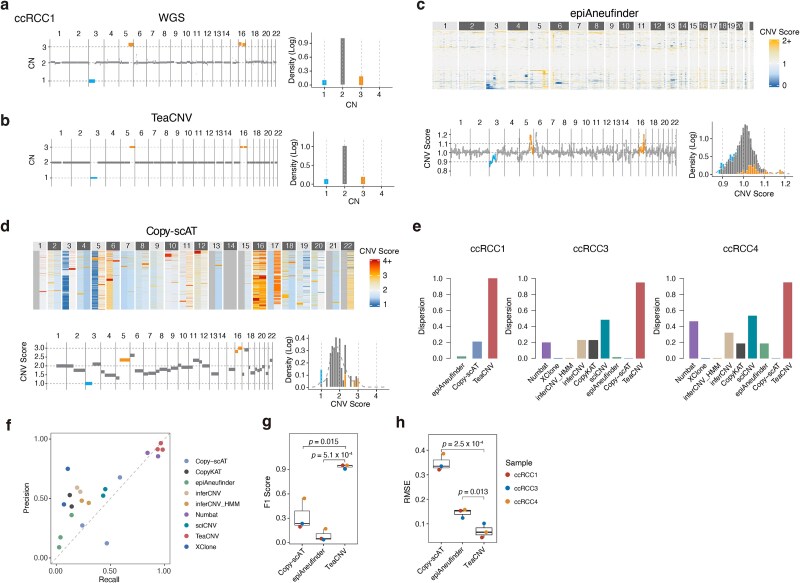
Performance evaluation of CNV detection methods. (a) Integer CN profiles derived from bulk WGS (left) and the corresponding genome-wide distribution of absolute CN states (right) for ccRCC1. Significantly altered regions are highlighted in blue (CN loss) and orange (CN gain). (b) Clonal integer CN profiles inferred by TeaCNV from chromatin accessibility data (left) and the genome-wide distribution of estimated integer CN states (right) for ccRCC1. (c, d) Relative CN profiles (top) and genome-wide distributions of relative CNV scores (bottom) estimated by (c) epiAneufinder and (d) Copy-scAT for ccRCC1. Blue (CN loss) and orange (CN gain) bars denote CNV regions identified by bulk WGS. (e) Dispersion scores of estimated CNs across genomic segments with distinct absolute CNs defined by bulk WGS. inferCNV-HMM indicates results from inferCNV in copy number state mode. Precision and recall (f), F1 score (g) for CNV detection across methods. Each dot represents one sample. *P*-values were calculated using Wilcoxon test. (h) Average deviation of inferred CN profiles from bulk WGS. RMSE: root mean square error.

In the scATAC-seq-only sample (ccRCC1), TeaCNV also demonstrated better performance in distinguishing chromosomal segments with distinct CN states compared with other scATAC-based approaches (Fig. 3a–d). We quantified the distinguishability of distinct integer CN states derived from bulk WGS data using dispersion score (Supplementary Methods). A higher score indicates less overlap among the inferred signal distributions of different integer CN states. TeaCNV achieved a higher average dispersion score (0.96 versus 0–0.51 for other approaches), reflecting clear separation of CN states (Fig. 3e). Across all samples, TeaCNV achieved 98.6% overall accuracy (precision: 93.0%, recall: 96.1%, F1 score: 0.92; detailed sample-level confusion matrix metrics are provided in Supplementary Fig. 7a) in CNV event identification, outperforming existing methods (Fig. 3f and g). Additionally, TeaCNV showed the lowest error in absolute CN profiles (average RMSE: 0.07, Fig. 3h, Supplementary Methods), indicating precise quantification of integer CN profiling. Furthermore, analysis of the detected CNV size distribution revealed a minimum event length of 3.2 Mb (Supplementary Fig. 7b).

### Detection of rare clone in clear cell renal cell carcinoma

**Figure 4:**
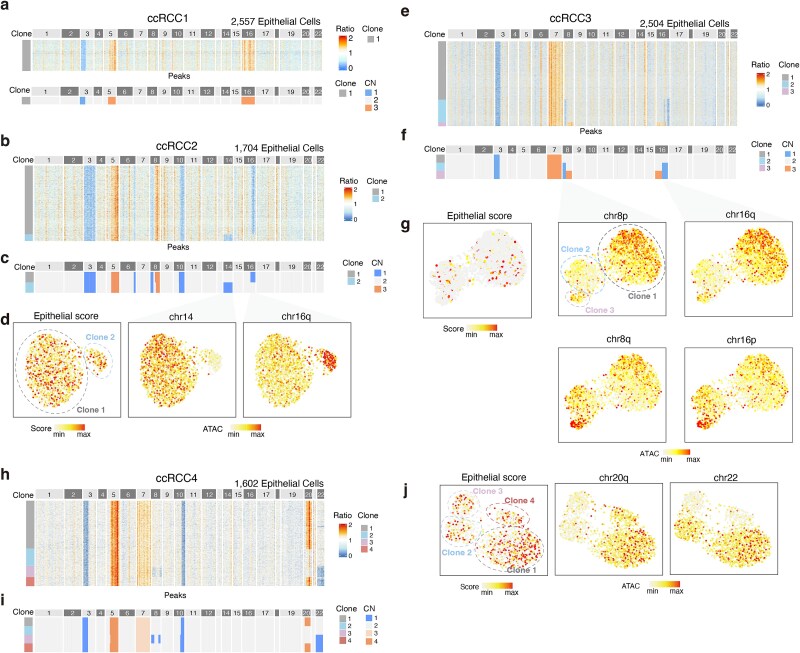
Resolving clonal substructures in ccRCC patients using scATAC-seq and scATAC&RNA-seq co-assay data. (a) Heatmap of copy ratio profiles in epithelial cells (top) and inferred clonal absolute CN profiles (bottom) for ccRCC1. Rows are annotated by clones. (b, c) TeaCNV results for ccRCC2: (b) copy ratio heatmap and (c) inferred clonal absolute CN profiles. (d) UMAP plots of epithelial cells from ccRCC2 colored by tumor activity scores (*EPCAM*, *KRT18*, *KRT8*, *KRT19)* and by ATAC signals from representative subclonal regions. (e, f) TeaCNV results for ccRCC3: (e) copy ratio heatmap and (f) inferred clonal absolute CN profiles. (g) UMAP plots of epithelial cells from ccRCC3 colored as in (d). (h, i) TeaCNV results for ccRCC4: (h) copy ratio heatmap and (i) inferred clonal absolute CN profiles. (j) UMAP plots of epithelial cells from ccRCC4 colored as in (d).

TeaCNV revealed distinct clonal architectures across the four ccRCC samples. While ccRCC1 displayed a monoclonal architecture, the other three cases showed polyclonal expansion dominated by a major subclone (Fig. 4). The clonal architectures and integer CN profiles remained robust regardless of the reference strategy used, whether using internal reference cell type (immune versus endothelial; Supplementary Fig. 8) or cross-sample references (Supplementary Fig. 9). Reliable reconstruction of clonal substructure required at least 20 reference cells, and performance remained robust across a range of reference cell fractions (Supplementary Fig. 10a and b). Additionally, at least 60% of epithelial cells were required to recapitulate the major population structure (Supplementary Fig. 10c).

Our analysis identified rare clones in samples with polyclonal substructures. In ccRCC2, two clones shared similar epithelial scores but diverged in chromatin accessibility patterns due to distinct absolute CNs at chr14 and chr16q. A rare clonal event involving chr14 loss was detected in 8.8% of tumor cells (Fig. 4b–d). For ccRCC3, TeaCNV resolved three subclones, including two minor subclones (clone2 and clone3: 32.59% combined) characterized by a 1-copy state at chr8p/16q, which is consistent with bulk WGS data (Figs 2c and 4e and f). A rare subclone (clone3: 5.19%) was further distinguished by 3 copies at chr8q/16p, accompanied by corresponding chromatin accessibility changes (Fig. 4g, Supplementary Fig. 11a). These rare clonal CNVs (gains of chr8q/16p) were also supported by inferCNV estimation but were undetectable in bulk WGS data due to limited cell representation (Fig. 2c and k). In ccRCC4, four subclones were stratified by distinct absolute CNs at chr20q and chr22 (Fig. 4h and i). Chr20q showed 4 copies in 66.4% of tumor cells and 2 copies in other tumor cells, consistent with the observation of an average of 3 copies from bulk WGS data (Figs 2d and f and 4i). Additionally, we identified a minor clonal event (chr22 loss) in 22.9% of tumor cells (clones 3 and 4). A rare clone (clone 4: 10.5%) displayed co-occurrence of chr20q gain (4 copies) and chr22 loss (1-copy) (Fig. 4i, Supplementary Fig. 11b). These events were also supported by inferCNV but undetected in bulk WGS (Fig. 2d and l).

### Dissecting genomic and epigenomic contributions to clonal heterogeneity in solid tumors

We applied TeaCNV to published scATAC-seq datasets [30] spanning pancreatic (PDAC), breast (BRCA), head and neck (HNSCC), colorectal (CRC), and ovarian (OV) cancers (Supplementary Fig. 12).

**Figure 5:**
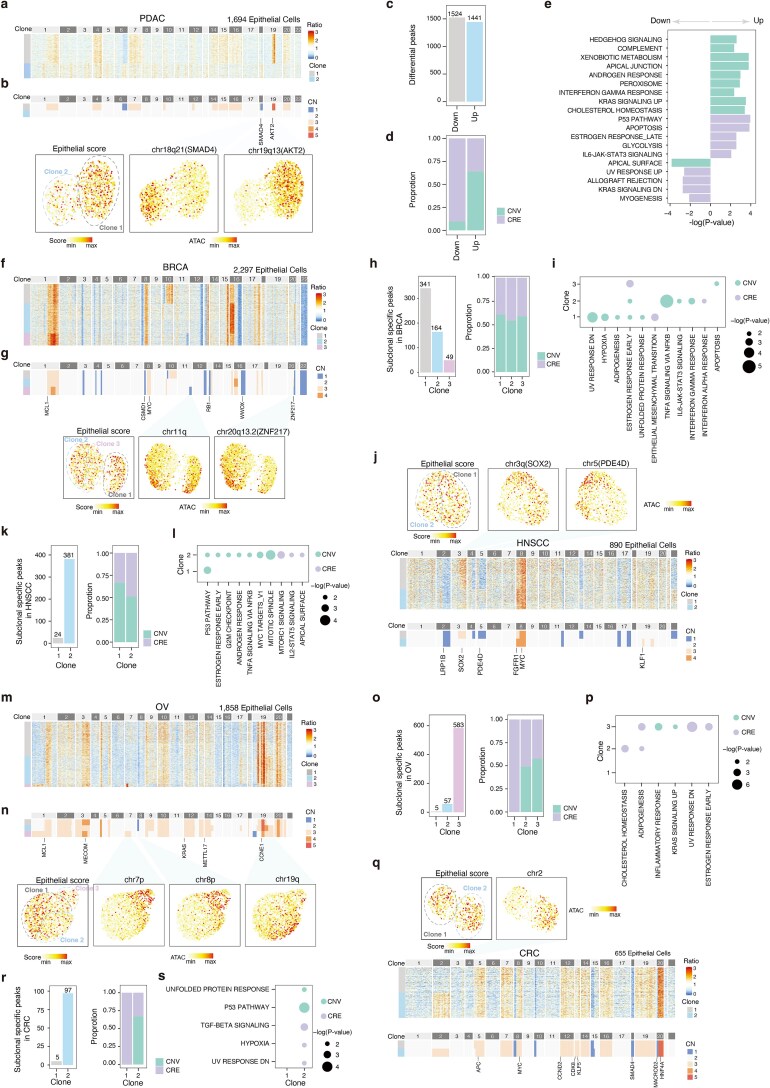
Clonal substructure in solid tumors. TeaCNV results for PDAC (a, b), BRCA (f, g), OV (m, n) samples: (a, f, m) copy ratio heatmaps and (b, g, n) inferred clonal absolute CN profiles (top) and UMAP plots (bottom) of epithelial cells colored by activity scores of tumor epithelial markers and ATAC-seq signals from representative subclonal regions. Known driver genes are highlighted in the clonal absolute CN profiles. (c) Number of differential accessibility peaks between aneuploid and diploid subpopulations in the PDAC sample. “Down” and “Up” denote peaks with decreased or increased accessibility in the aneuploid versus diploid subpopulation, respectively. (d) Proportion of differential peaks driven by CNVs and CREs in the PDAC sample. (e) Hallmark pathways significantly enriched by CNV-driven and CRE-regulated peak associated genes in PDAC. (j, q) TeaCNV results for HNSCC (j) and CRC (q) samples: UMAP plots (top) of epithelial cells colored by activity scores of tumor epithelial markers and ATAC-seq signals from representative subclonal regions, copy ratio heatmap (middle), and inferred clonal absolute CN profiles (bottom). Subclone-specific accessibility analysis for BRCA (h, i), HNSCC (k, l), OV (o, p), CRC (r, s) samples: (h, k, o, r) the number of subclone-specific peaks (left), and the proportion of CNV-driven and CRE-regulated peaks (right); (i, l, p, s) hallmark pathways significantly enriched by CNV-driven and CRE-regulated subclone-specific peak associated genes.

Analysis of 1694 PDAC epithelial cells revealed 1 aneuploid and 1 diploid subpopulation, with the diploid subpopulation exhibiting lower epithelial scores (Fig. 5a and b). TeaCNV identified PDAC driver events, including *AKT2* amplifications and *SMAD4* deletions, with distinct chromatin accessibility patterns between subpopulations (Fig. 5b) [49]. Differential peak analysis showed that 64% of upregulated peaks in the aneuploid subpopulation were CNV-driven (i.e. peaks located within CNV regions), enriching hallmark pathways including xenobiotic metabolism, apical junction organization, KRAS signaling, and cholesterol homeostasis (Fig. 5c–e). CRE-regulated peaks (i.e. peaks located outside CNV regions) in the aneuploid subpopulation were associated with activation of P53 signaling, apoptosis, and glycolysis (Fig. 5e).

All epithelial cells in the BRCA, HNSCC, OV, and CRC tumor samples were aneuploid and exhibited polyclonal architectures. In the BRCA sample, truncal CNVs involved driver genes (*MCL1*, *MYC*, *CSMD1*, *RB1*, and *WWOX,*  Fig. 5f and g) [50]. Subclonal CNV events (chr11q, chr20q13.2/*ZNF217*) delineated three subclones with similar epithelial scores but distinct ATAC signals (Fig. 5g). More than half of the subclonal-specific peaks were CNV-driven, dominating the phenotypic diversity across subclones (Fig. 5h and i). In the HNSCC sample, truncal CNVs involved known driver genes, including *LRP1B*, *FGFR1*, and *MYC* (bottom of Fig. 5j) [51]. Subclonal events harboring cancer genes (*SOX2, PDE4D*, and *KLF1)* drove clone-specific accessibility patterns (Fig. 5j). CNV-driven peaks mediated P53 pathway dysregulation in both subclones and specifically proliferation activation in clone 2 (G2M checkpoint, MYC, and mitotic spindle), highlighting the major contribution of CNVs to phenotypic divergence across subclones (Fig. 5k and l).

In the OV sample, truncal CNVs involved *MCL1*, *MECOM*, *KRAS*, *METTL17*, and *CCNE1* [52] (Fig. 5m and n). Despite comparable proportions of CNV-driven and CRE-regulated peaks within each subclone, pathway diversity across subclones was primarily CRE-mediated (cholesterol homeostasis in clone 2, adipogenesis/UV response in clone 3, Fig. 5o and p). Similarly, in the CRC tumor sample, subclonal CNVs altered chromatin accessibility patterns, but most enriched pathways were CRE-regulated (Fig. 5q–s) [53]. Moreover, TeaCNV correctly inferred a diploidy copy number profile across epithelial cells using immune cells as the reference population in normal CRC (Supplementary Fig. 12f). It further recovered major CNV patterns and clonal substructures across multiple solid tumors, even when using ccRCC-derived non-epithelial cells as a cross-dataset reference (Supplementary Fig. 13). These findings demonstrate the complex interplay of genomic and epigenomic mechanisms in driving tumor subclonal heterogeneity.

## Discussion

We developed TeaCNV, a computational framework that enables robust estimation of absolute CN profiles and clonal architecture directly from scATAC-seq data, without requiring matched bulk DNA sequencing. TeaCNV resolves CN states beyond simple gain or loss, achieving an absolute CN quantification error of less than 0.5, as validated by simulated and bulk WGS data. Additionally, TeaCNV improves the resolution of tumor heterogeneity in scATAC-seq datasets, particularly for detecting rare clonal CNVs with a prevalence below 10%, which are often undetectable in bulk WGS data (Supplementary Fig. 14). Finally, TeaCNV facilitated the decoupling of genomic and epigenomic contributions to phenotypic heterogeneity, revealing the mechanisms underlying diverse phenotypes across tumor clones.

ScATAC-seq-based CNV detection faces fundamental limitations, including mappability bias, GC content effects, extreme data sparsity, and CRE-related biases [35, 54]. While existing methods address GC content and mappability issues, they remained constrained by sparsity and CRE biases, limiting detection to relative gains or losses at arm-level genomic scales rather than absolute CNs quantification [33, 34]. Although the scDNA-seq-based method Alleloscope [35] can be applied to scATAC-seq data to estimate allelic copy number, its dependence on matched bulk DNA-seq data poses a major limitation.

TeaCNV addresses potential mappability bias by removing low-quality peaks, a filtering strategy (e.g. excluding ENCODE Blacklist regions) similar to other methods. To minimize inadvertent discarding signals from biologically meaningful regions, we apply this filtering conservatively while aiming to balance signal detection with reduced systematic bias. In addition, TeaCNV calculates inferred-to-reference ratios, which normalize GC-related fluctuations and are independent of GC content (Supplementary Fig. 15). By merging adjacent peaks into genomic windows and aggregating sparse signals within cell subpopulations, TeaCNV reduces the biases introduced by data sparsity and CRE effects. In ccRCC samples, differential CREs explained less than 10% of the CNV variance and over 50% of differential CREs were located outside CNV regions, indicating that TeaCNV effectively reduces confounding effects (Supplementary Fig. 16).

Applied to 6 cancer types, TeaCNV revealed polyclonal architectures in 77.78% of cases, demonstrating its broad utility across solid tumors. By enabling precise deconvolution of clonal substructure and absolute CN quantification, TeaCNV supports accurate functional dissection of large-scale CNV regions as well as the contribution to phenotypic diversity [10, 55–57]. We observed numerous differential chromatin accessibility loci across intra-tumor subclones, which underlie phenotypic variations. These differential loci arise through distinct mechanisms: one is driven by CNVs that directly alter chromatin accessibility within affected regions (accounting for a substantial fraction of variance), and another involves CREs located outside CNV regions, which are largely independent of CNVs (Supplementary Fig. 16). Therefore, the complex interplay between genomic and epigenomic mechanisms driving tumor subclonal heterogeneity can be dissected from scATAC-seq using the absolute CN inference. Furthermore, TeaCNV facilitates distinguishing the distinct oncogenic roles of co-altered genes in single-cell multi-omics (scATAC&RNA-seq co-assay) sequencing data, where chromosomal dosage effects can be discerned from large-scale gene expression changes associated with absolute copy number.

Four considerations guide the application of TeaCNV. First, it performs optimally on subclones composed of sufficiently homogeneous cells, as sparse data limits the ability to call single-cell CNVs—a limitation shared by all scATAC-seq-based approaches. Tumor cell lines or samples with continuous clonal gradients or high subclonal diversity (e.g. those containing numerous rare subpopulations) may yield unstable estimates. Based on benchmarking with simulated data, the minimal subclonal size for reliable detection is 20 cells. Accordingly, TeaCNV is optimized for dissecting clonal architecture and genotype-epigenotype coupling at the subclonal level, rather than at the level of individual cells. Second, robust reference cell selection is critical, particularly for highly malignant samples lacking internal normal cells. TeaCNV ideally requires reference cells from the matched sample with at least 20 cells. Estimation using cross-dataset referencing can also capture the main clonal substructure and CNVs present in the tumor cell population, but may introduce deviations in subclonal CNV events; thus, intra-dataset or batch-matched referencing is preferred over cross-dataset approaches for superior robustness. Third, although TeaCNV performs robustly for broad and large sub-arm-level CNVs, its resolution for focal events is inherently limited by the intrinsic sparsity of scATAC-seq data. Very short genomic intervals often lack sufficient accessible chromatin coverage and signal-to-noise ratio for sensitive CNV detection and precise breakpoint resolution. Consequently, focal amplifications and deletions, including potentially clinically actionable events, may be missed or not resolved as independent CNVs (Supplementary Fig. 7c–e). This limitation becomes particularly evident for smaller focal events, with sensitivity declining markedly below 10 Mb and approaching zero for events smaller than 2 Mb. Lastly, TeaCNV currently infers total CN and does not resolve allele-specific alterations (e.g. loss of heterozygosity or copy-neutral events), which may limit analyses of allelic phenomena such as tumor suppressor loss and immune evasion. Efforts are underway to extend it by incorporating phased genetic variation and/or joint modeling with multi-omic data, which may enable allele-specific CNV inference and further enhance its utility for analyzing complex tumor genomes.

Tumor plasticity arises from aberrant activation of transcriptional programs caused by genetic and non-genetic mechanisms [58–60]. With advancements in single-cell sequencing technologies, simultaneous profiling of the epigenome and transcriptome at single-cell resolution is now possible [61, 62]. TeaCNV bridges this gap in single-cell multiomics by connecting clonal genotypes to epigenomic and transcriptomic phenotypes. By enabling absolute CNV analysis in any scATAC-seq dataset, TeaCNV empowers systematic exploration of how genomic instability shapes epigenetic diversity—a critical factor underlying therapeutic resistance and metastatic progression.

**Key Points**

- We developed TeaCNV, a computational framework that infers absolute, genome-wide copy number profiles directly from scATAC-seq, without requiring matched DNA baselines, and robustly determines genome ploidy.
- TeaCNV deconvolves high-resolution clonal substructure, reconstructing CNV-driven polyclonal architectures in most tumors and detecting rare subclones with high sensitivity.
- Across simulated, in-house and public datasets from six cancer types, TeaCNV outperforms existing scATAC- and scRNA-based methods, recovering rare CNVs and clinically relevant driver alterations missed by bulk WGS.
- TeaCNV delineates subclonal CNV architectures and links this genotypic diversity to epigenotypic and transcriptional changes, such as CNV-associated chromatin remodeling, transcriptional heterogeneity, and dysregulated signaling pathways within tumor clones.

## Supplementary Material

Supplementary_materials_bbag265

## Data Availability

The scATAC-seq, scATAC&RNA-seq co-assays and bulk WGS validation data of ccRCC can be accessed in Zenodo at https://doi.org/10.5281/zenodo.14190637. scATAC-seq data of BRCA, PDAC, HNSCC, CRC, and OV were downloaded from Gene Expression Omnibus (GEO) with accession number GSE240822.

## Code availability

The TeaCNV algorithm is available at https://github.com/ShaojunLab/TeaCNV. The analysis scripts used to reproduce results and simulation scripts included in the paper are available at https://github.com/ShaojunLab/TeaCNVanalysis.
