# Supplementary material for: Inferring tumor absolute copy number and clonal substructure from single-cell chromatin accessibility
Source: Brief Bioinform. 2026 May 27;27(3):bbag265. doi: 10.1093/bib/bbag265 (PMC13215597; doi:10.1093/bib/bbag265)
Supplement: Supplementary_materials_bbag265 [file supplementary_materials_bbag265.zip › Supplementary Files.docx]

**Supplementary Material**

**Inferring tumor absolute copy number and clonal substructure from single cell chromatin accessibility**

Ying Wang^#1,2^, Yuhao Deng^#3^, Hang Li^1^, Xinbao Yin^4^, Yanru Zhang^3^, Yurong Chen^5^, Min Zhang^3^, Xin Wang^3^, Zhizhuo Cao^3^, Shaojun Zhang^1^*

1. Guangdong Academy of Medical Sciences and Medical Research Institute, Guangdong Provincial People’s Hospital (Guangdong Academy of Medical Sciences), Southern Medical University, Guangzhou, P.R. China.
2. Center for Endemic Disease Control, Chinese Center for Disease Control and Prevention, Harbin Medical University, Harbin, P.R. China.
3. Institute of Precision Medicine, The First Affiliated Hospital, Sun Yat-Sen University, Guangzhou, P.R. China
4. Department of Urology, The Affiliated Hospital of Qingdao University, Qingdao, Shandong, China.
5. Department of Gynecological Radiotherapy, Harbin Medical University Cancer Hospital, Harbin, China

* Corresponding authors: Shaojun Zhang, Medical Research Institute, Guangdong Provincial People’s Hospital (Guangdong Academy of Medical Sciences), Southern Medical University, Guangzhou, P.R. China. E-mail: szhang5@outlook.com.

# These authors contributed equally

**Supplementary Methods**

**Optimizing absolute copy number estimation**

As described in the main text, the expected relative copy ratios $\mu={\{\mu}_{q}|q\in Q\}$ for integer CN states $Q=\{1,2,\ldots,I\}$ were first estimated by maximum likelihood under the mixture model. In an ideal setting, these expected ratios should be approximately linearly spaced with respect to CN state, with a constant increment corresponding to a one-copy change. To formalize this, we defined the difference between adjacent states as:

$$\Delta_{q}=\mu_{q}-\mu_{q-1} (q=2,\ldots,I)$$

Theoretically, $\Delta_{q}$ is expected to be constant and less than 1 across different integer CN states $q$ (e.g., $\Delta_{q}\approx0.5$ corresponds to a change of 1-copy relative to diploid). However, due to uneven sequencing coverage or technical noise, biases in the $\mu_{q}$ estimation may result in non-constant $\Delta_{q}$values across $q$ (**Supplementary Fig. 17a**). To mitigate such bias and enforce a more coherent spacing between CN states, we performed a Δ-based regularization and model-selection procedure.

We first generated candidate $\Delta$ values by: 1) iterating over all unique observed $\Delta_{q} (q=2,\ldots,I)$; 2) considering 2$\cdot\Delta_{q}$ for $\Delta_{q}<0.3$ to avoid overfitting; 3) considering $\frac{1}{2}\cdot\Delta_{q}$ for$\Delta_{q}>0.6$ to avoid underfitting.

For the *i*-th candidate $\Delta_{i}$, the expected copy ratios are defined as:

$$\mu^{\left( i \right)}=\{\mu_{q}^{\left( i \right)}=\mu_{1}+\left( q-1 \right)\cdot\Delta_{i}|q\in Q\}$$

We then calculated the Akaike Information Criterion (AIC) value for each candidate $\mu^{\left( i \right)}$.

$${AIC}_{i}=2k_{i}-ln\left( \hat{L}^{(i)} \right)$$

Where $k_{i}$ is the maximum integer CN state in $\mu^{\left( i \right)}$ and $\hat{L}^{(i)}$ is the likelihood of the observed data given $\mu^{\left( i \right)}$. The optimal absolute CN estimation for each subpopulation is determined by the candidate $\mu^{\left( i \right)}$ with the minimum AIC value (**Fig. 1c**).

**Tumor subclone partitioning**

To avoid potential over-clustering of tumor cell subpopulations, we defined an initial adjacency matrix $A$ to indicate whether two subpopulations can be merged into a single clone.

$$A_{i,j}=\left\{ \begin{aligned} 1, i=j \\ 0,i\neq j \end{aligned} \right.$$

Here, $A_{i,j}=1$ indicates that cell subpopulation $s_{i}$ and $s_{j}$ can be merged, while 0 indicates they cannot.

To update the adjacency matrix $A$, we computed the average peak signals for each subpopulation ($s_{i})$:

$${Y^{'}}_{s_{i}}=\left[ \bar{Y}'_{(1, s_{i})},\ldots,\bar{Y}'_{(n,s_{i})} \right]$$

where $n$ is the number of peaks.$\bar{Y}'_{(k, s_{i})}$ represents the average value of the $k$-th peaks across cells in subpopulation $s_{i}$.

Next, we calculated the peak signal ratio between subpopulation $s_{i}$ and $s_{j}$:

$$R_{s_{i},s_{j}}=\frac{{Y^{'}}_{s_{i}}}{{Y^{'}}_{s_{j}}}$$

Using this ratio, we performed genome segmentation as described previously. For each segmented region, we compared the peak signal distributions between subpopulation $s_{i}$ and $s_{j}$ using a two-sided t-test with $p$-values adjusted by the Benjamini-Hochberg method.

If any segmented region has an adjusted $p$-value < 0.05 and the integer CN estimates of $s_{i}$ and $s_{j}$ differ, we set:

$$A_{i,j}= A_{j,i}=0$$

Otherwise, we updated the matrix as:

$$A_{i,j}= A_{j,i}=1$$

The final subclonal partitioning was derived from the updated adjacency matrix. Integer CN profiles for each subclone were then estimated using the same method as previously described. We computed the overall ploidy of each subclone as the weighted sum of the estimated integer CN states:

$$ploidy=\sum_{j=1}^{l} w_{j}\cdot\hat{q}_{j}$$

**Scoring the confidence of copy number profiling of tumor subclone**

We proposed a scoring metric to evaluate the reliability of integer CN profiles for tumor subclones. Based on the hypothesis that observed relative copy ratios should closely match the expected ratios for integer CN states in a homogeneous subclone (**Supplementary Fig. 17b**), we first calculated the mean squared error (MSE) between observed and expected copy ratios for each subclone:

$$MSE=\frac{1}{l}\sum_{i=1}^{l} \left( R_{i}-\mu_{i} \right)^{2}$$

where *l* is the total number of segmented chromosomal regions. $R_{i}$ is the observed relative copy ratio of segment $i$. $\mu_{i}$ is the expected copy ratio of segment $i$, determined by its assigned integer CN state.

Theoretically, the absolute deviation $\left| R_{i}-\mu_{i} \right|$ should be smaller than $\Delta$ (i.e., the expected relative copy ratio difference caused by a single-copy state change). Therefore, the theoretical upper bound of MSE is less than 1, because $\Delta<1$.

To further account for the variability of chromosomal segmental lengths, we also computed the genomic proportion of regions explained by the expected copy ratios within an allowed error $d (d\ll\Delta)$:

$$F=\sum_{q\in Q} \left( \frac{\sum_{i\in\{l|\left| R_{l}-\mu_{q} \right|<d\}} {length}_{i}}{L} \right)$$

where $L$ is the total length of the genome. ${length}_{i}$ is the length of segment *i*. $Q$ is the set of integer CN states. We set$d=0.1$ in this study.

In addition, excessive segmentation (large $l$) or underestimation of $\Delta$ may lead to overestimation of $F$ and ploidy, as well as underestimation of MSE due to overfitting. To balance these factors, we integrated MSE and F using a weighted parameter $\beta$ which penalizes $\Delta$, $l$ and ploidy, to define the final score:

$$score=F\cdot\left( -logMSE \right)\cdot\beta$$

where:

$\beta=\frac{\delta_{\Delta}}{log\left( 1+l \right)\cdot e^{ploidy-2}}$, $\delta_{\Delta}=\left\{ \begin{aligned} \Delta, if \Delta<0.3 \\ 1, otherwise \end{aligned} \right.$

Overall, a higher score indicates a more reliable and homogeneous subclone, reflecting a smaller MSE, a larger F and balanced segmentation.

**Evaluating performance of copy number estimation**

To evaluate CNV detection performance, we used all CNVs identified by bulk WGS as ground truth, regardless of event size, and calculated precision, recall, accuracy (ACC), and F1 score based on the overlap between predicted and true CNVs. The genome was divided into 100-kb bins, excluding those overlapping breakpoints in either ground truth or inferred results. We defined true positive (TP) as bins with CNVs present in both the ground truth and the inferred results, false positive (FP) as bins with CNVs present in the inferred results but absent in the ground truth, false negative (FN) as bins with CNVs in the ground truth but not detected in the inferred results, and true negative (TN) as bins without CNVs in either (**Supplementary Fig. 7a)**. Precision, recall, ACC and F1 score were then calculated as:

$$precision= \frac{TP}{TP+FP}$$

$$recall= \frac{TP}{TP+FN}$$

$$ACC= \frac{TP+TN}{TP+TN+FP+FN}$$

$$F1= \frac{2\cdot precision\cdot recall}{precision+recall}$$

Additionally, to evaluate the accuracy of TeaCNV in reconstructing polyclonal substructure, we calculated the BACC across all subclones to measure the consistency between the estimated polyclonal substructure and the ground truth. For a given subclone $c$, we computed the BACC as:

$${BACC}_{c}=\frac{1}{2}\left( {recall}_{c}+{specificity}_{c} \right)=\frac{1}{2}\left( \frac{{TP}_{c}}{{TP}_{c}+{FN}_{c}}+\frac{{TN}_{c}}{{TN}_{c}+{FP}_{c}} \right)$$

Where ${TP}_{c}$, ${FN}_{c}$, ${TN}_{c}$, ${FP}_{c}$ denote the true positive, false negative, true negative, and false positive cells for subclone $c$, respectively.

The overall balanced accuracy of polyclonal substructure within a tumor cell population was calculated as the weighted average across subclones:

$${BACC}_{weighted}=\frac{\sum_{c=1}^{K} n_{c}\cdot{BACC}_{c}}{\sum_{c=1}^{K} n_{c}}$$

where $n_{c}$ denotes the number of cells in subclone $c$. This weighted formulation ensures that both dominant and rare subclones contribute proportionally to the final performance metric, reflecting the accuracy of subclone label prediction across the entire polyclonal substructure.

Since TeaCNV reports CNVs at clonal level, we averaged the precision, recall and F1 score across the subclones derived from each sample. For epiAneufinder and Copy-scAT, CNV events were considered detected at the bulk level if they were identified in a specific proportion of cells, ranging from 10% to 100% in 10% increments. The average precision, recall, and F1 score were then calculated across these varying cell proportion thresholds.

To evaluate the accuracy of inferred CN profiles, we calculated the deviation of estimations from WGS using the root mean square error (RMSE) metric:

$$RMSE= \sqrt{\frac{1}{n}\cdot\sum_{i=1}^{n} \left( {CN}_{i}-{CN}_{i}^{true} \right)^{2}}$$

Here, ${CN}_{i}$ represents the inferred integer CN for bin *i* and ${CN}_{i}^{true}$ is the true integer CN derived from WGS.

To quantify how well distinct integer CN states can be distinguished, we proposed a dispersion score to measure the separation between inferred signal distributions corresponding to different integer CN states. Ideally, the distributions of inferred values for genomic regions with different absolute CN states should not overlap. A higher dispersion score indicates less mixing of CN state signals and greater confidence in CN estimation. It is calculated as the sum of non-overlapping areas between the probability density functions (PDFs) of each pair of adjacent integer CN states ($s,s+1$), normalized to the range [0, 1] (**Supplementary Fig. 17c**):

$$dispersion= \sum_{s=1}^{I-1} \left( \frac{\int_{A_{1}} p_{s}\left( x \right)dx+\int_{A_{2}} p_{s+1}\left( x \right)dx}{2} \right)$$

$$A_{1}=\{x|p_{s}\left( x \right)\neq0 \cap p_{s+1}\left( x \right)=0\}$$

$$A_{2}=\{x|p_{s}\left( x \right)=0 \cap p_{s+1}\left( x \right)\neq0\}$$

Here, $I$ represents the maximum absolute CN states. $p_{s}\left( x \right)$ is the probability density of the inferred values for the genomic regions with integer CN *s* in WGS data.

For TeaCNV, the inferred value is output integer CN profiles. For other methods, the inferred value is the estimated CNV score. A larger dispersion suggests a better ability to distinguish distinct absolute CN states, reflecting a more accurate estimation of CN states.

**Simulation of scATAC-seq data**

To benchmark CNV detection performance, we generated simulated scATAC-seq datasets with well-defined ground truth using simATAC (v1.0.0), an R package that models signal distributions based on real scATAC-seq data. As references for parameterization, we used all immune cells from both ccRCC3 and ccRCC4 to capture the distribution of non-malignant (normal) cells, and four tumor subclones from ccRCC3 together with subclone 2 from ccRCC4 to represent malignant subclonal distributions.

Each simulation run was initialized with an independent random seed, and the number of cells in each subclonal population was determined according to the designed composition. This process generated peak-by-cell count matrices exhibiting realistic sparsity and library-size heterogeneity consistent with empirical distributions modeled by simATAC.

We simulated datasets with varying population size by fixing 2,000 normal reference cells and varying inferred cell populations from 50 to 10,000 (50, 100, 200, …, 1,000, 2,000, 5,000, and 10,000). For each inferred population size, we modeled five clonal architectures: monoclonal, biclonal, triclonal, tetraclonal, and pentaclonal. In polyclonal settings (except monoclonal), the subclone with a 10% frequency was designated as the rare clone. Clonal and subclonal CNVs were kept consistent with the true CNV profiles identified in the corresponding real tumor subclones, ensuring biological realism in the simulation.

To examine performance under different degrees of genomic alteration, we fixed the inferred population size at 2,000 cells and varied CNV burden from 10% to 60%, categorized into low (≤30%), intermediate (30–40%), and high (≥40%) burden groups. Across all simulation conditions, independent replicates were generated using different random seeds, resulting 680 datasets in total (summarized in **Supplementary Table S1**). Although summary statistics such as total span and size-bin counts may coincide across replicates, the genomic coordinates and copy number states of CNV segments are independently generated in each dataset.

To further assess TeaCNV performance across broad and focal CNVs, we generated 100 additional simulated scATAC-seq datasets, each containing 2,000 inferred cells and 2,000 normal reference cells, with genome-wide focal and broad CNV events. Focal CNVs were defined as events spanning less than 50% of a chromosome arm, and broad CNVs as larger events. Across the simulated genomes, ~9% of genomic regions were affected by focal CNVs, with event sizes ranging from 1.1 kb to 40.7 Mb, whereas ~19% of genomic regions were affected by broad (arm-level) CNVs (**Supplementary Fig. 2c)**. Notably, although very short focal CNVs were included in the simulated ground truth, the intrinsic sparsity of scATAC-seq data caused variance over short genomic intervals to be confounded with true CNV signals, thereby limiting the resolution for very short focal CNVs in both simulated scATAC-seq profiles and downstream CNV inference from such data.

We applied TeaCNV, epiAneufinder and Copy-scAT to each simulated dataset for benchmarking. For epiAneufinder and Copy-scAT, cells with fewer than 5,000 fragments were excluded after applying a per-cell fragment-count filter. For comparison across methods with different segmentation outputs, we partitioned the genome into fixed 100-kb bins. We then mapped the ground-truth CNV segments and each method’s predicted CNV segments to these bins and labeled each bin as CNV-positive if it overlapped any CNV segment (ground truth or predicted). We then computed TP, FP, FN, TN counts at the bin level, and derived precision, recall, and F1 scores. TeaCNV calls were computed per subclone and averaged, whereas epiAneufinder/Copy-scAT calls were thresholded by the fraction of cells (10%–100% in 10% steps) and averaged across thresholds. Summary statistics are reported in **Supplementary Table S2**. Computational runtime was recorded for all analyses on a standardized computing environment (28-core CPU, 256 GB RAM).

**Simulation of scDNA-seq data**

To evaluate the capability of bulk WGS data to detect CNV signals from rare subclones, we simulated idealized scDNA-seq data based on the CNV profiles of subclones 1-3 from the ccRCC4 sample, as inferred by TeaCNV, which served as the ground-truth single-cell CNV map. The simulated tumor population consisted of 2,000 cells with subclone proportions of 5:4:1, corresponding to a 10% frequency for the rare subclone.

Under an idealized high-coverage single-cell DNA sequencing scenario without technical bias, we modeled the fragment counts for genomic bin i as Poisson-distributed with an expected coverage λ = 200, scaled according to the relative copy number of the corresponding genomic region. To mimic amplification efficiency, alignment bias, and GC-content variability, multiplicative Gaussian noise (mean = 1, variance = 10) was introduced.

In total, we simulated ~14,000 genomic bins (average bin length ≈ 1.2 kb) to generate the scDNA-seq count matrix, and similarly simulated 2,000 normal cells as reference. After normalization, the mean bin-wise coverage across tumor and normal cells was computed to generate pseudobulk DNA profiles. The relative CNV ratio of aggregated DNA-seq data was then obtained by taking the tumor-to-normal coverage ratio (**Supplementary Fig. 14b**).

**Differential chromatin accessibility analysis**

We performed a differential chromatin accessibility analysis on PDAC, BRCA, HNSCC, OV and CRC samples. We identified differential peaks across subclones within individual sample using “FindAllmarkers” function in Signac, with thresholds of absolute log2fc > 0.25 and adjusted p < 0.01. The identified subclonal-specific peaks were classified as CNV-driven if they were located within the corresponding subclonal CNV regions; otherwise, they were classified as CRE-regulated.

**Gene set enrichment analysis**

For the identified clone-specific peaks, we obtained the associated genes using the “ClosestFeature” function in Signac. Gene set enrichment analysis was then performed based on hallmark gene sets from MSigDB. Significant pathways were defined as those with $p$-values < 0.01.

**Supplementary Figures**

**
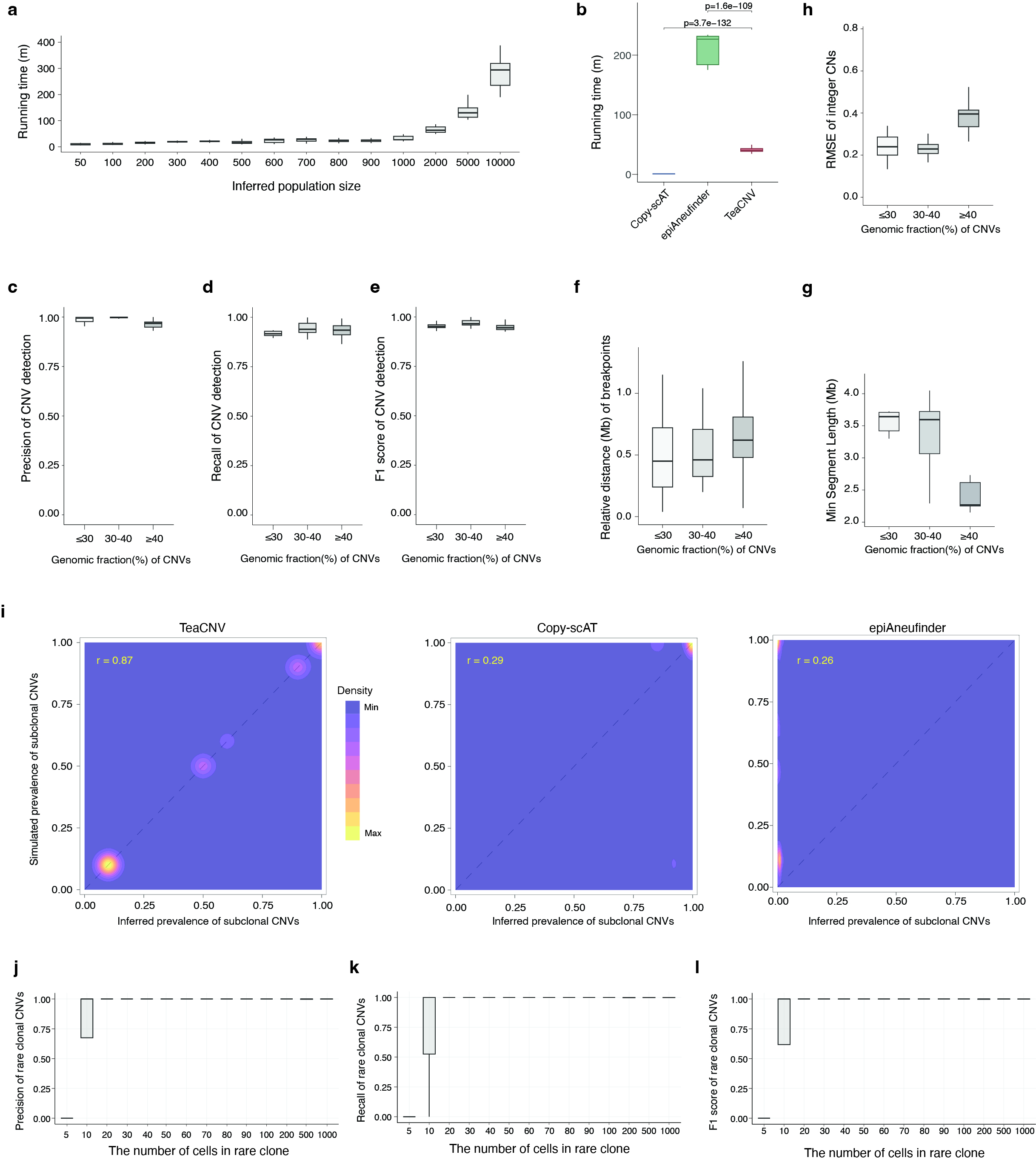
**

**Supplementary Figure 1.** Benchmarking of TeaCNV based on simulated scATAC-seq datasets. (**a**) Running time of TeaCNV across varying population sizes. (**b**) Running time on 2,000 cells across different methods. CNV detection performance by TeaCNV across different genome fractions of CNVs based on precision (**c**), recall (**d**) and F1 score (**e**). (**f**) Relative deviation of chromosomal breakpoints inferred by TeaCNV compared to the ground truth across different genome fractions of CNVs. (**g**) Minimum detectable CNV segment length across varying genomic fractions of CNVs. (**h**) RMSE of inferred CNs by TeaCNV across varying genomic fractions of CNVs. (**i**) Correlation between inferred and simulated subclonal CNV frequencies for TeaCNV, Copy-scAT and epiAneufinder. Precision (**j**), recall (**k**) and F1 score (**l**) for rare clonal CNV detection by TeaCNV based on simulated scATAC-seq datasets, with rare clone sizes ranging from 5 to 1,000 (corresponding to total population sizes ranging from 50 to 10,000).

**ALT TEXT:** Graphs summarizing benchmarking of TeaCNV on simulated scATAC-seq datasets, with panels showing runtime across population sizes and methods, CNV detection performance (precision, recall, F1), breakpoint deviation, CN RMSE and minimum detectable CNV segment length across varying CNV genome fractions, correlations between inferred and simulated subclonal CNV frequencies, and accuracy metrics for rare clonal CNV detection over a wide range of clone sizes.


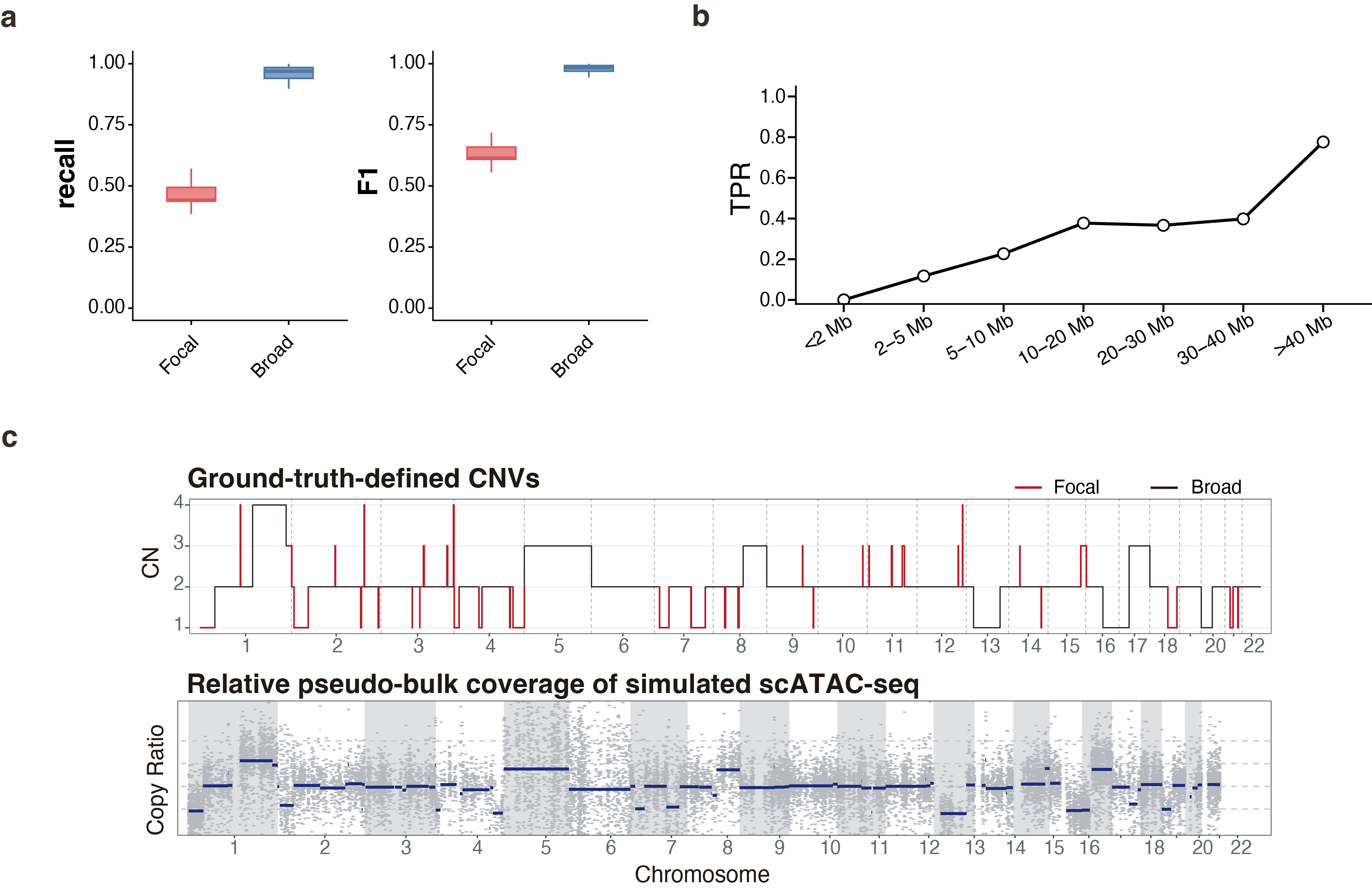


**Supplementary Figure 2.** TeaCNV performance on simulated scATAC-seq data with focal and broad CNVs. (**a**) Recall and F1 score of TeaCNV for focal and broad CNVs. (**b**) Sensitivity of TeaCNV for focal CNVs stratified by event size. TPR: True positive rate. (**c**) Simulated genome-wide CNV landscape and the corresponding pseudo-bulk scATAC-seq coverage profile. Top, ground-truth CNV events across chromosomes, including focal CNVs (red) and broad CNVs (black). Bottom, relative pseudo-bulk coverage of the simulated scATAC-seq data across the same genome, showing the mean copy-ratio signal over inferred cells on defined segments.

ALT TEXT: Graphs show TeaCNV performance on simulated scATAC-seq data containing focal and broad CNVs. Panel a shows boxplots of recall and F1 score, indicating better performance for broad CNVs than for focal CNVs. Panel b shows true positive rate for focal CNVs across size intervals, with sensitivity increasing as CNV size increases. Panel c displays genome-wide ground-truth CNV events across chromosomes, with focal events in red and broad events in black, together with the corresponding pseudo-bulk copy-ratio profile.


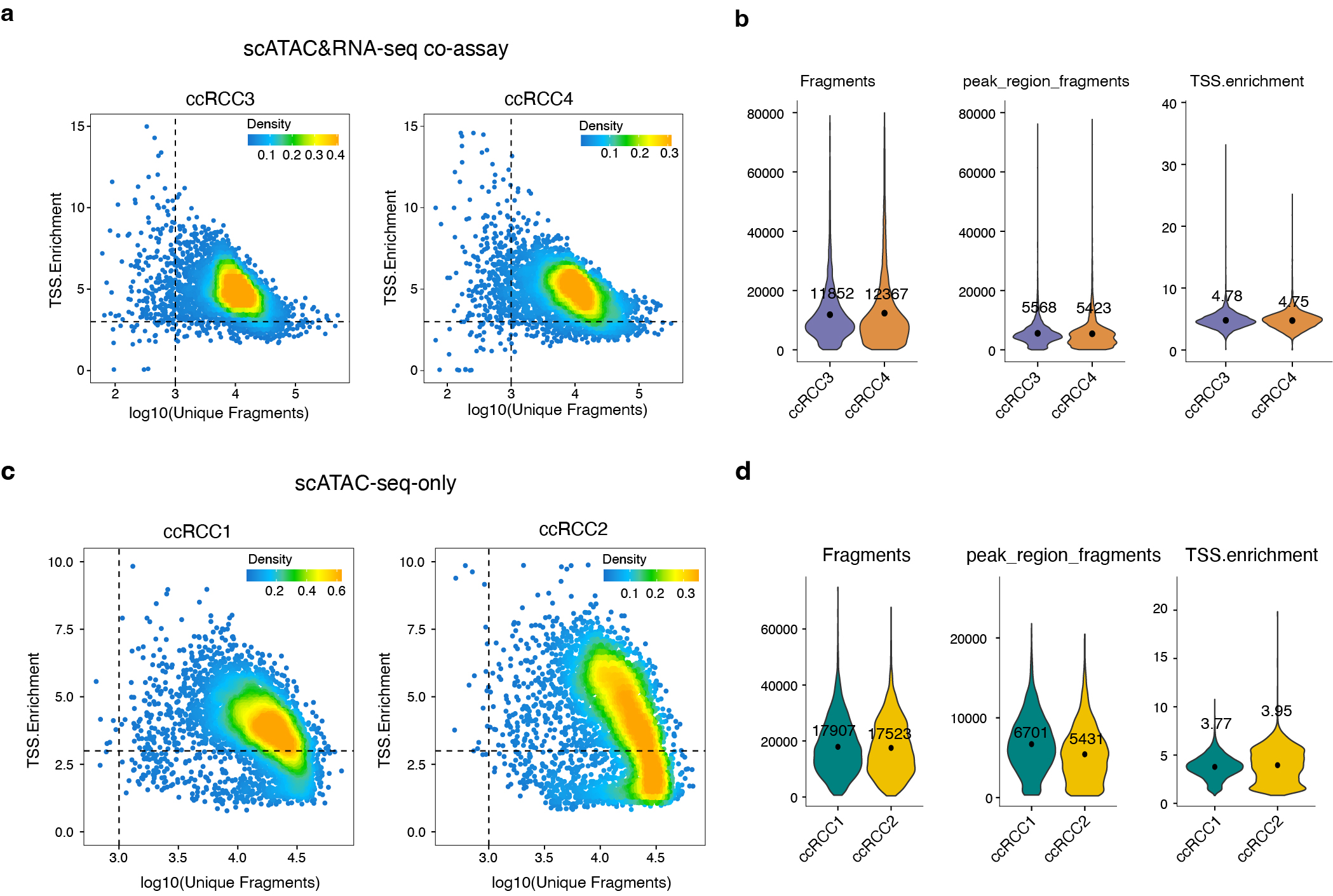


**Supplementary Figure 3.** Data quality of scATAC&scRNA-seq co-assayed and scATAC-seq only data for ccRCC patients. (**a**) and (**c**) The number of unique ATAC-seq fragments in each single cell (each dot) compared with TSS enrichment of all fragments in that cell. Dashed lines represent cutoffs for filtering high-quality cells. Dots are colored by density. (**b**) and (**d**) Violin plots show the distribution of total fragment counts, the number of fragments located in peaks, and TSS enrichment values across individual samples.

**ALT TEXT:** Scatter and violin plots assessing scATAC&scRNA co-assay and scATAC-only data quality in ccRCC patients, showing relationships between per-cell ATAC fragment counts and TSS enrichment with filtering cutoffs, and distributions of total fragments, in-peak fragments and TSS enrichment across samples.


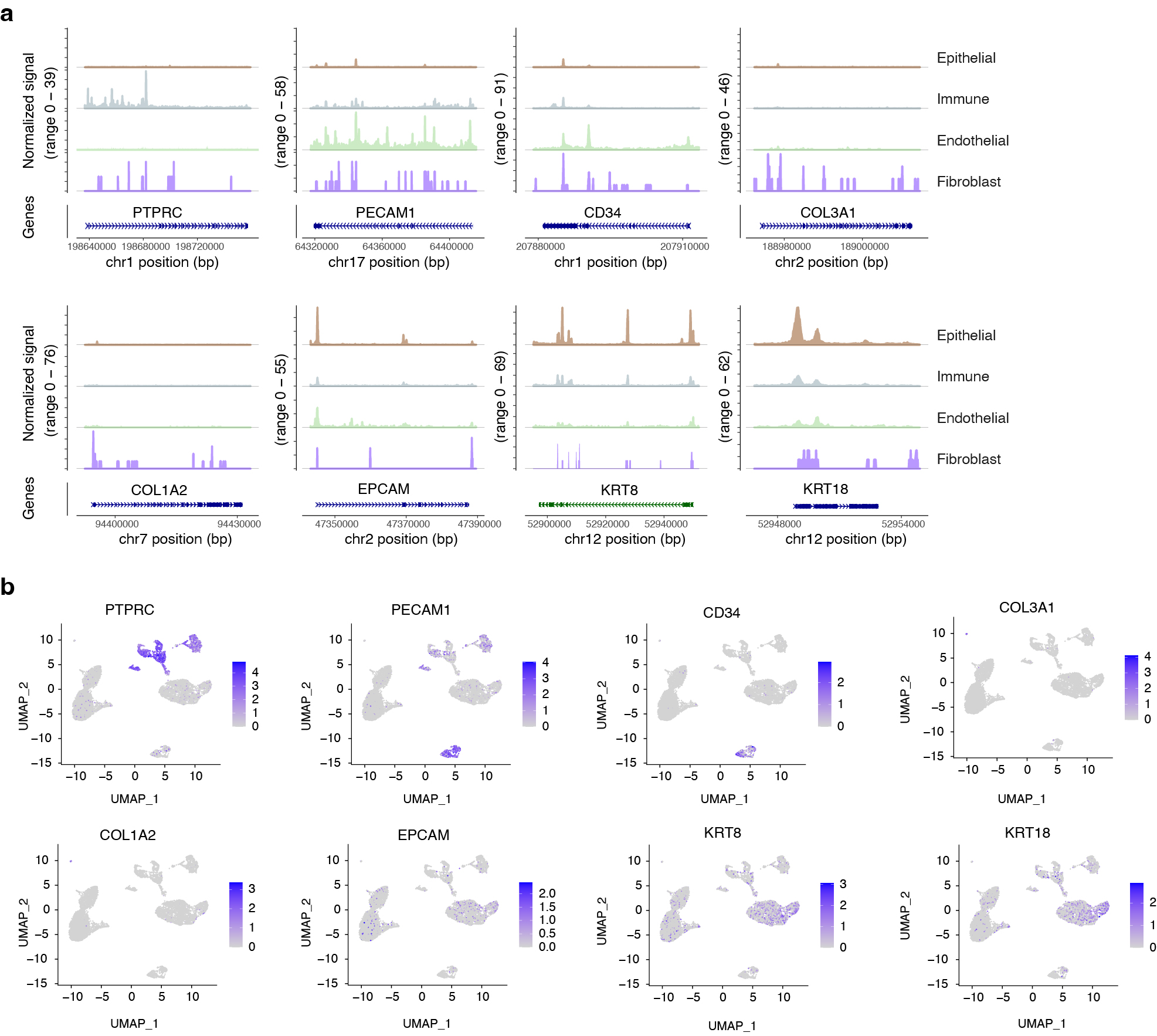


**Supplementary Figure 4.** Cell annotation of scATAC&RNA-seq co-assayed data from two ccRCC patients. (**a**) Genome track of aggregate scATAC-seq signals across cells near the cell-type marker locus. Tracks are colored by cell type: epithelial, immune, endothelial, and fibroblast cells. (**b**) Expression levels of cell-type marker genes from scRNA-seq data.

**ALT TEXT:** Genome track and expression plots illustrating cell-type annotation in scATAC&RNA co-assay data from two ccRCC patients, with aggregate scATAC-seq signals near marker loci and scRNA-seq marker gene expression profiles distinguishing epithelial, immune, endothelial and fibroblast cells.


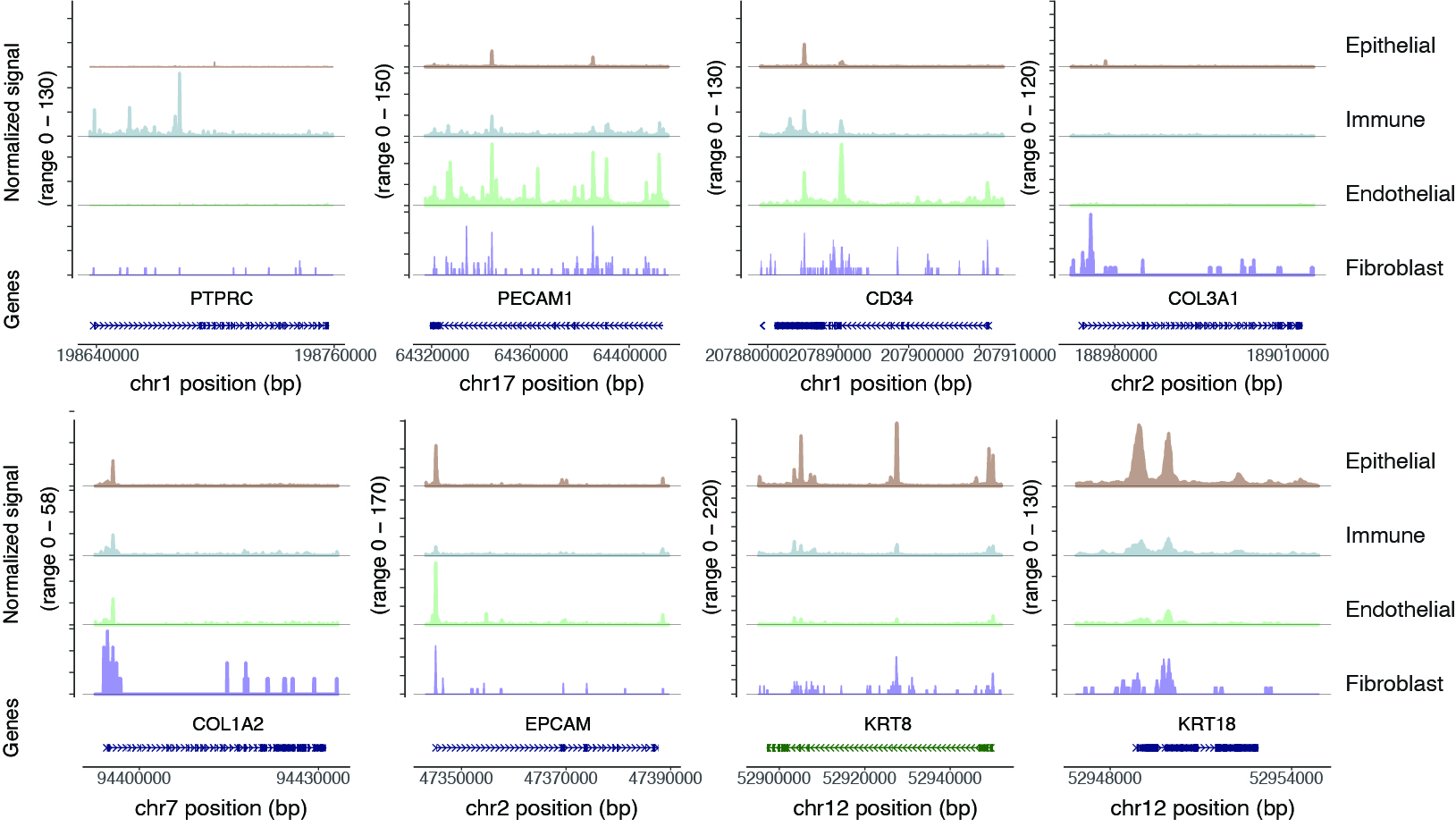


**Supplementary Figure 5.** Genome track of aggregate scATAC-seq signals across cells near the cell-type marker locus for scATAC-seq-only samples. Tracks are colored by cell type: epithelial, immune, endothelial, and fibroblast cells.

**ALT TEXT:** Genome tracks showing aggregate scATAC-seq accessibility near cell-type marker loci in scATAC-only samples, with signal profiles colored by epithelial, immune, endothelial and fibroblast cell types.


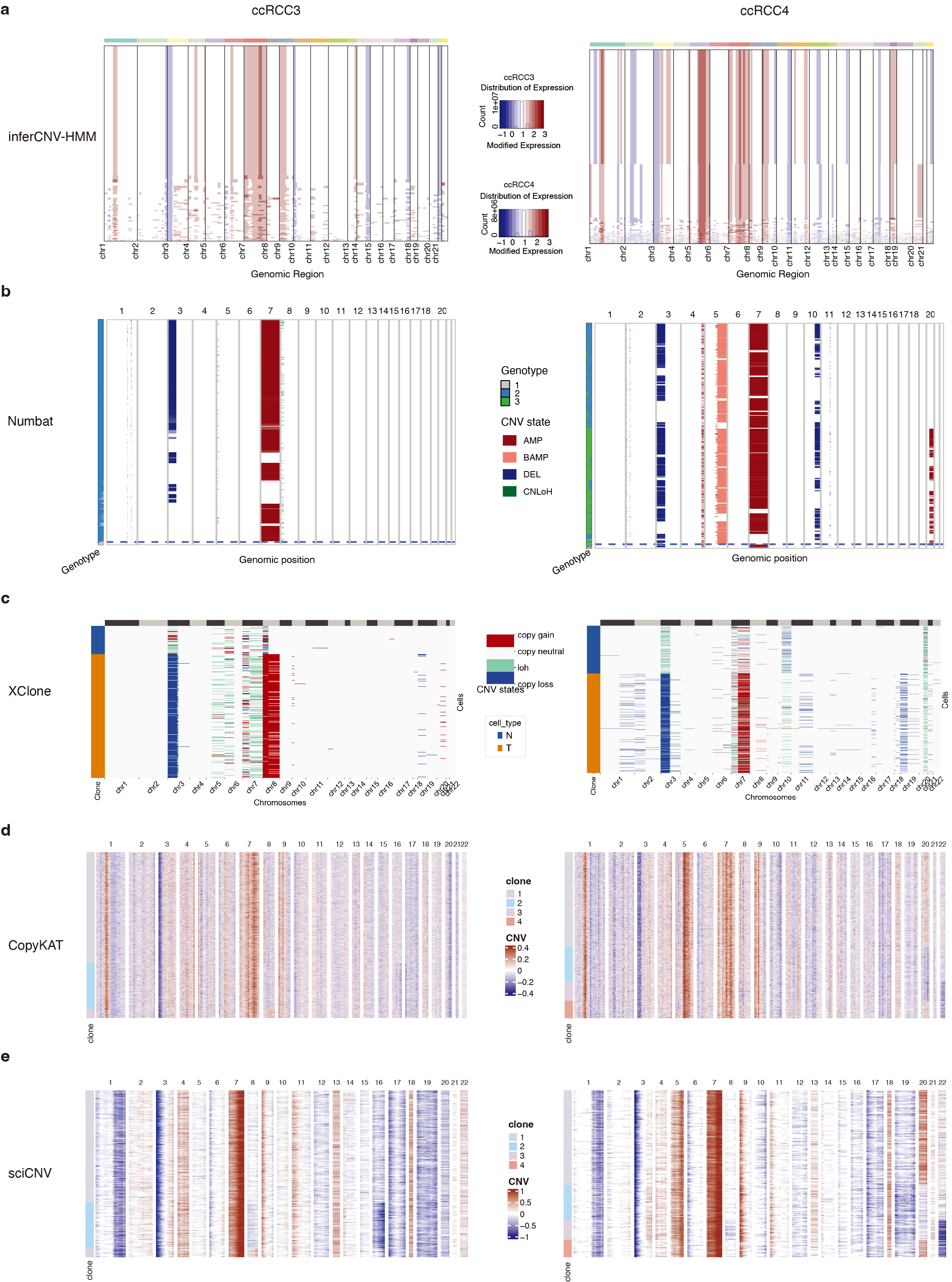


**Supplementary Figure 6.** Inferred CNV profiles from scRNA-seq data of ccRCC3 and ccRCC4 using (**a**) inferCNV in copy number state mode (inferCNV-HMM), (**b**) Numbat, (**c**) Xclone, (**d**) CopyKAT and (**e**) sciCNV.

**ALT TEXT:** Inferred CNV profiles from scRNA-seq data of ccRCC3 and ccRCC4 using inferCNV-HMM, Numbat, Xclone, CopyKAT and sciCNV.


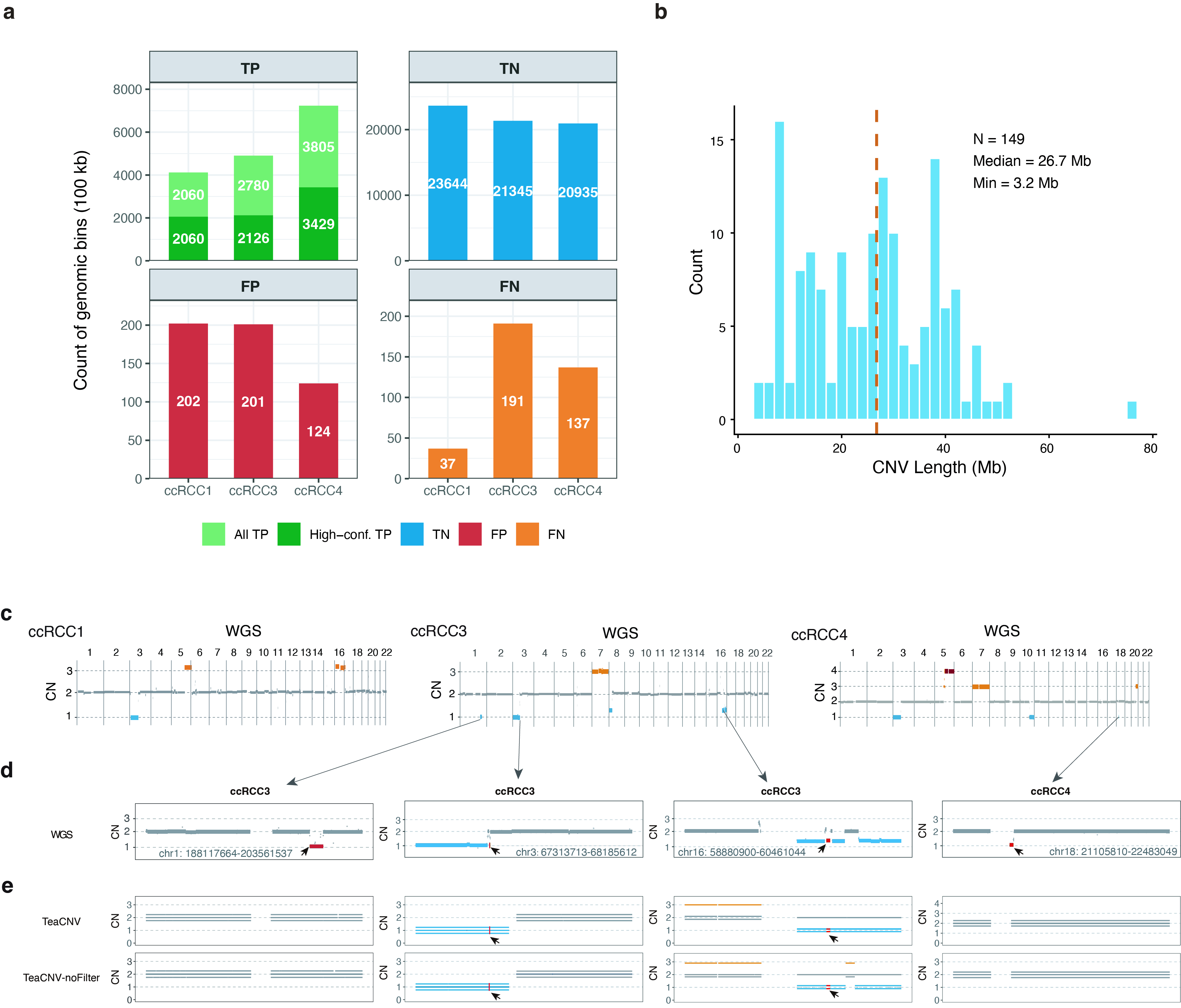


**Supplementary Figure 7.** Detailed statistics for CNV detection across ccRCC samples using TeaCNV. (**a**) Bar plots illustrate the breakdown of TP, TN, FP, and FN for the three ccRCC samples. The TP panel (top-left) utilizes a stacked bar representation: the dark green segment ('High-conf. TP') denotes strict, high-confidence consensus calls (used for precision calculation), while the light green segment extends to the total count ('All TP'), representing the comprehensive set of detected events (used for recall calculation). All were assessed at the 100 kb genomic bin level. (**b**) Size distribution of detected CNV events for all ccRCC samples. The vertical dashed line indicates the median CNV size (26.7 Mb), and the total number of CNV segments (N) is annotated. (**c**) Genome-wide ground-truth CNV profiles inferred from matched WGS in ccRCC samples. (**d**) Zoomed-in views of representative focal CNV events from the WGS profiles. (**e**) TeaCNV-inferred CNV states in the corresponding focal regions, shown with segment-size filtering enabled (top) or disabled (bottom).

**ALT TEXT:** Supplementary Figure 7 summarizes CNV detection in three ccRCC samples using TeaCNV. Panel a shows bar plots of true positives, true negatives, false positives, and false negatives at 100 kb bin resolution, with true positives divided into high-confidence and total calls. Panel b shows the size distribution of detected CNV events, with the median size marked by a dashed line. Panel c shows genome-wide ground-truth CNV profiles from matched WGS. Panel d shows enlarged views of representative focal CNV events. Panel e shows TeaCNV-estimated CNV states in the same focal regions, comparing results with segment-size filtering enabled versus disabled.


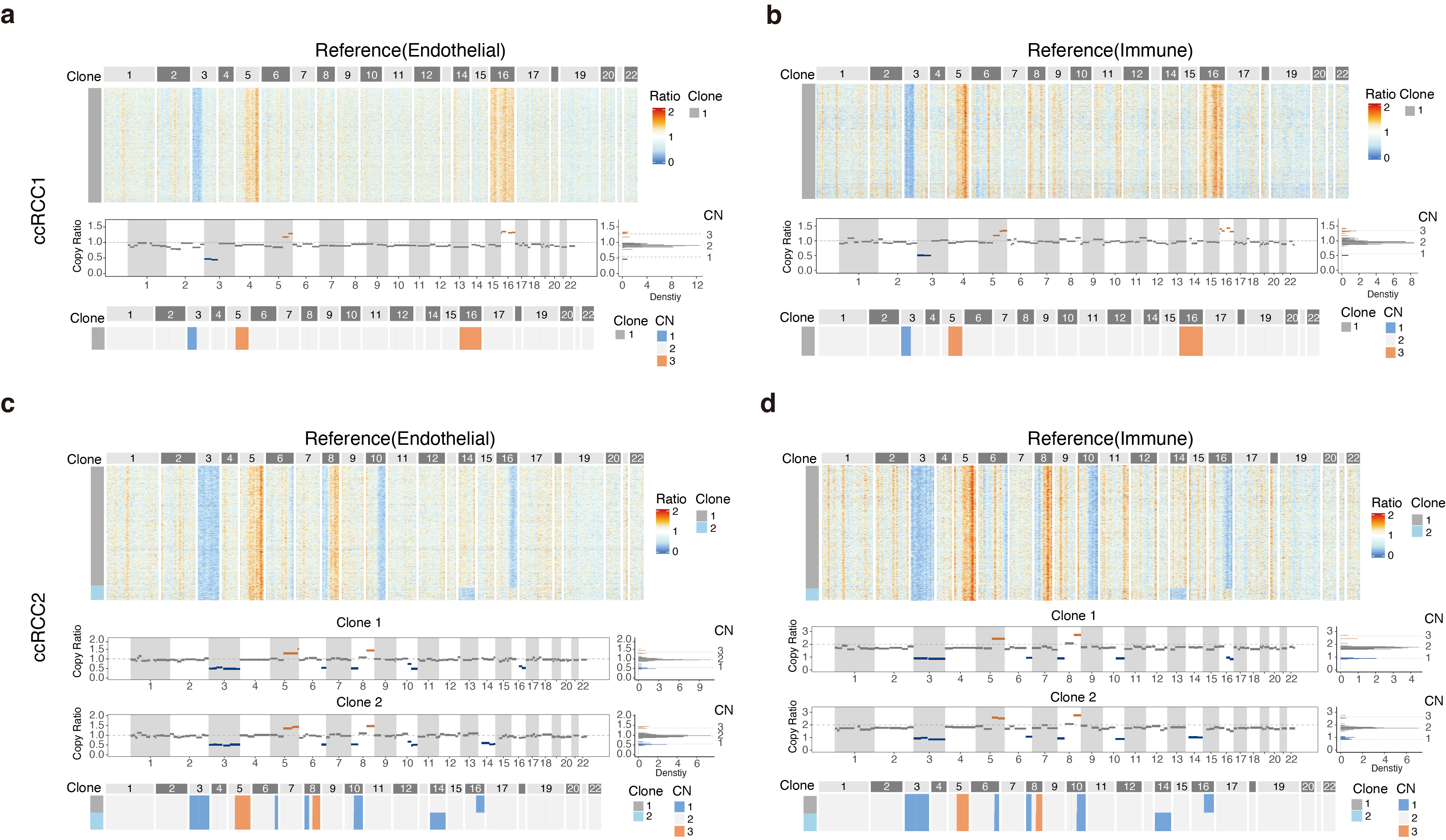


**Supplementary Figure 8.** Validation of TeaCNV robustness using different internal reference cells. (**a-b**) Absolute CNs of epithelial cells from ccRCC1 were inferred using endothelial cells (**a**) and immune cells (**b**) as references. (**c-d**) Absolute CNs of epithelial cells from ccRCC2 were inferred using endothelial cells (**c**) and immune cells (**d**) as references. The panels include: a heatmap displaying the copy ratio of epithelial cells relative to the reference (top), a genome-wide segmentation of copy ratios for clones (middle left) with the corresponding copy ratio histogram (middle right), and a heatmap of inferred clonal absolute CN profiles (bottom).

**ALT TEXT:** Heatmaps and genome-wide copy-number summaries demonstrating TeaCNV robustness in ccRCC1 and ccRCC2 when using different internal reference cell types, showing epithelial cell copy ratios relative to endothelial or immune references, segmented clone-level copy-ratio profiles with corresponding histograms, and inferred clonal absolute copy-number profiles.


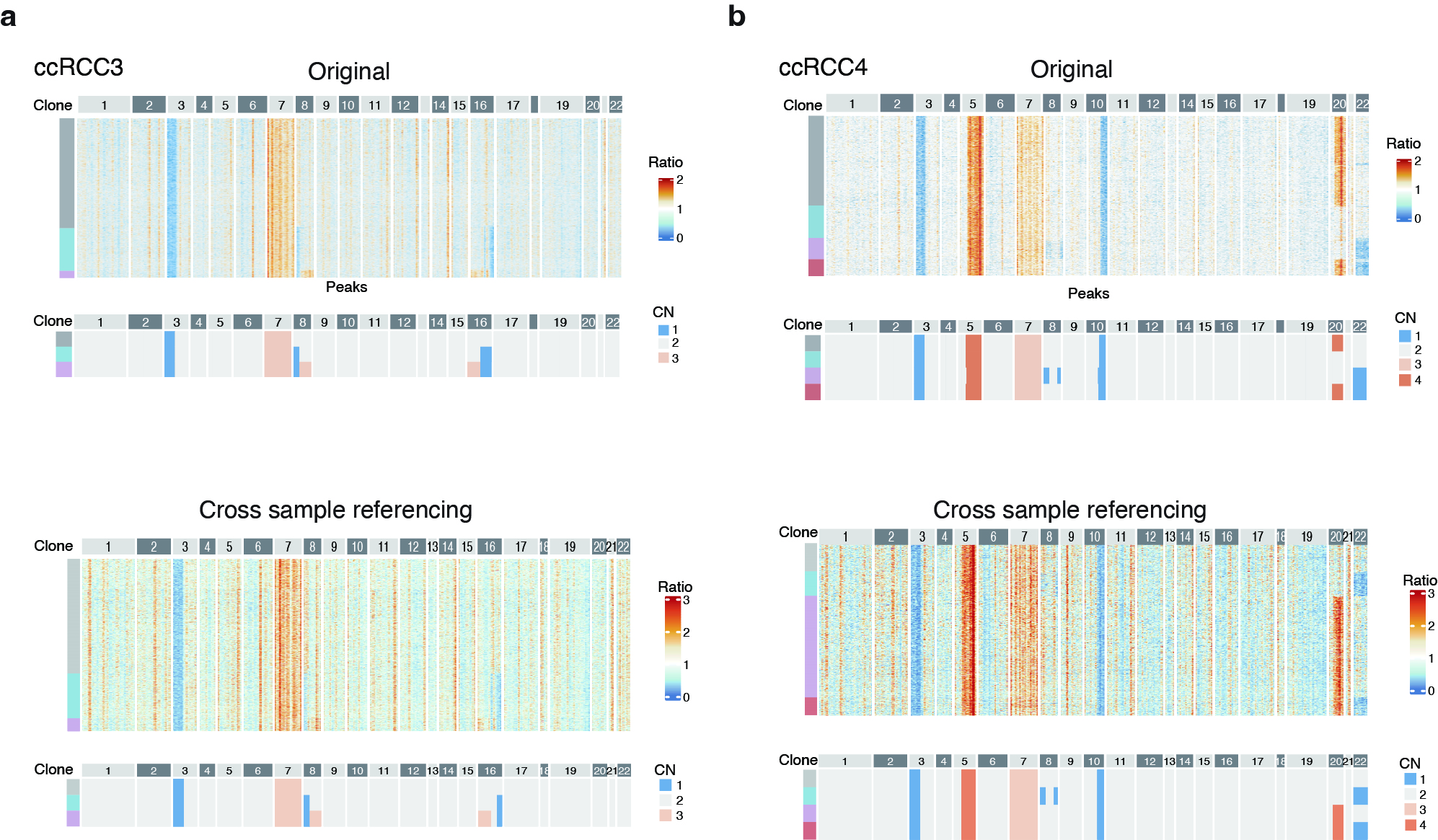


**Supplementary Figure 9.** Feasibility evaluation for cross-sample reference cell requirements. (a) Original estimation (top) and ccRCC4 reference-based estimation (bottom) of ccRCC3 by TeaCNV. (b) Original estimation (top) and ccRCC3 reference-based estimation (bottom) of ccRCC4 by TeaCNV.

**ALT TEXT:** Heatmaps demonstrating cross-sample referencing for ccRCC3 and ccRCC4 with the same experimental batch is feasible, showing epithelial cell copy ratios and inferred clonal absolute copy-number profiles for original estimation and cross-sample reference-based estimation.


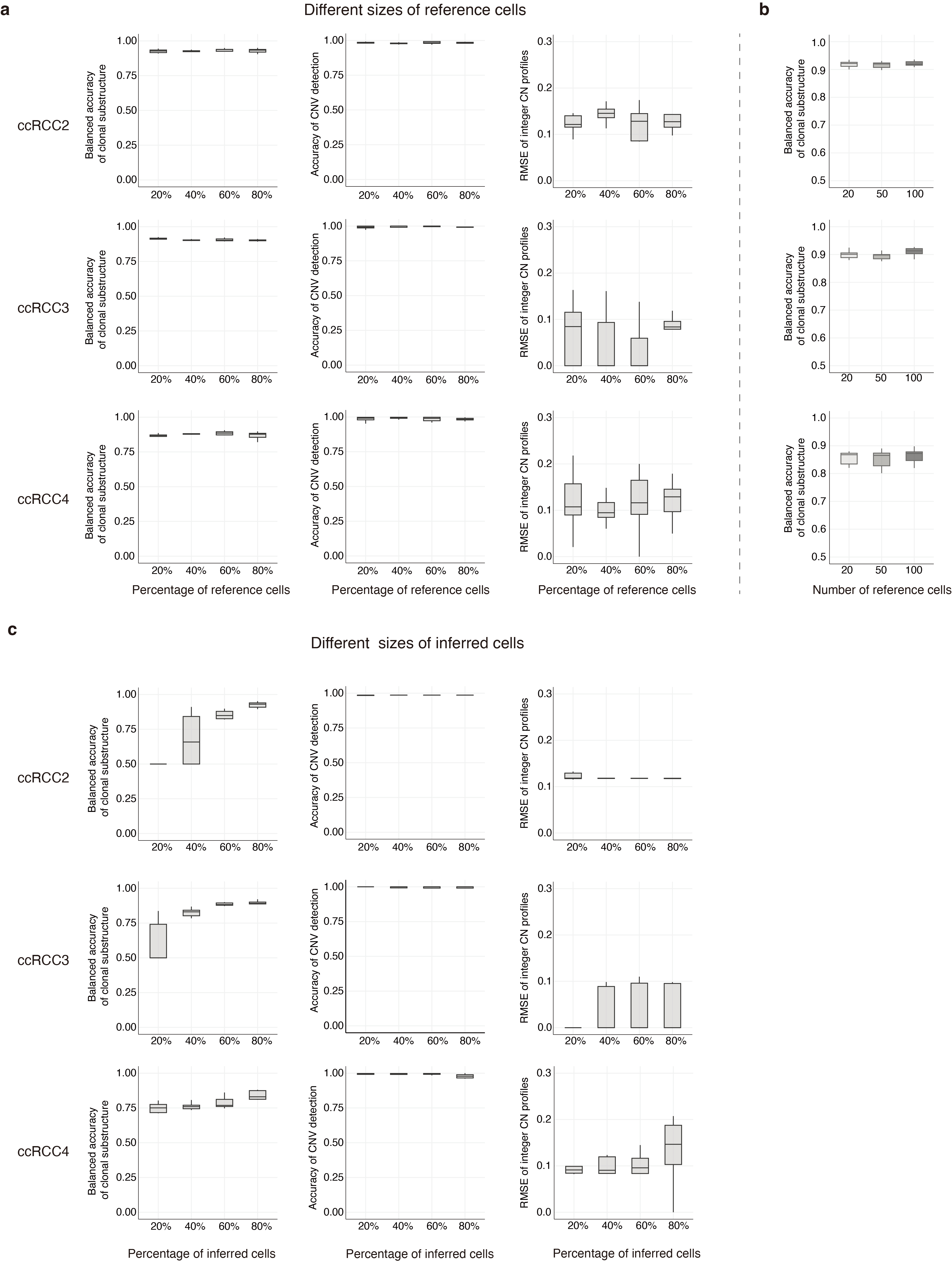


**Supplementary Figure 10.** Robustness of TeaCNV estimation in ccRCC samples. (**a**) Evaluation of TeaCNV performance using different percentages of reference cells from ccRCC samples. From left to right: clonal substructure consistency (compared to the full reference), accuracy of CNV detection, and error of integer CN profiles. (**b**) Evaluation of clonal substructure reconstruction under different numbers of reference cells. (**c**) Evaluation of TeaCNV performance using different percentages of epithelial cells from ccRCC samples. From left to right: clonal substructure consistency (compared to the full epithelial cells), accuracy of CNV detection, and error of integer CN profiles.

**ALT TEXT:** Graphs assessing the robustness of TeaCNV in ccRCC samples, showing how varying percentages and numbers of reference or epithelial cells influence clonal substructure consistency relative to the full dataset, CNV detection accuracy, and errors in inferred integer copy-number profiles.


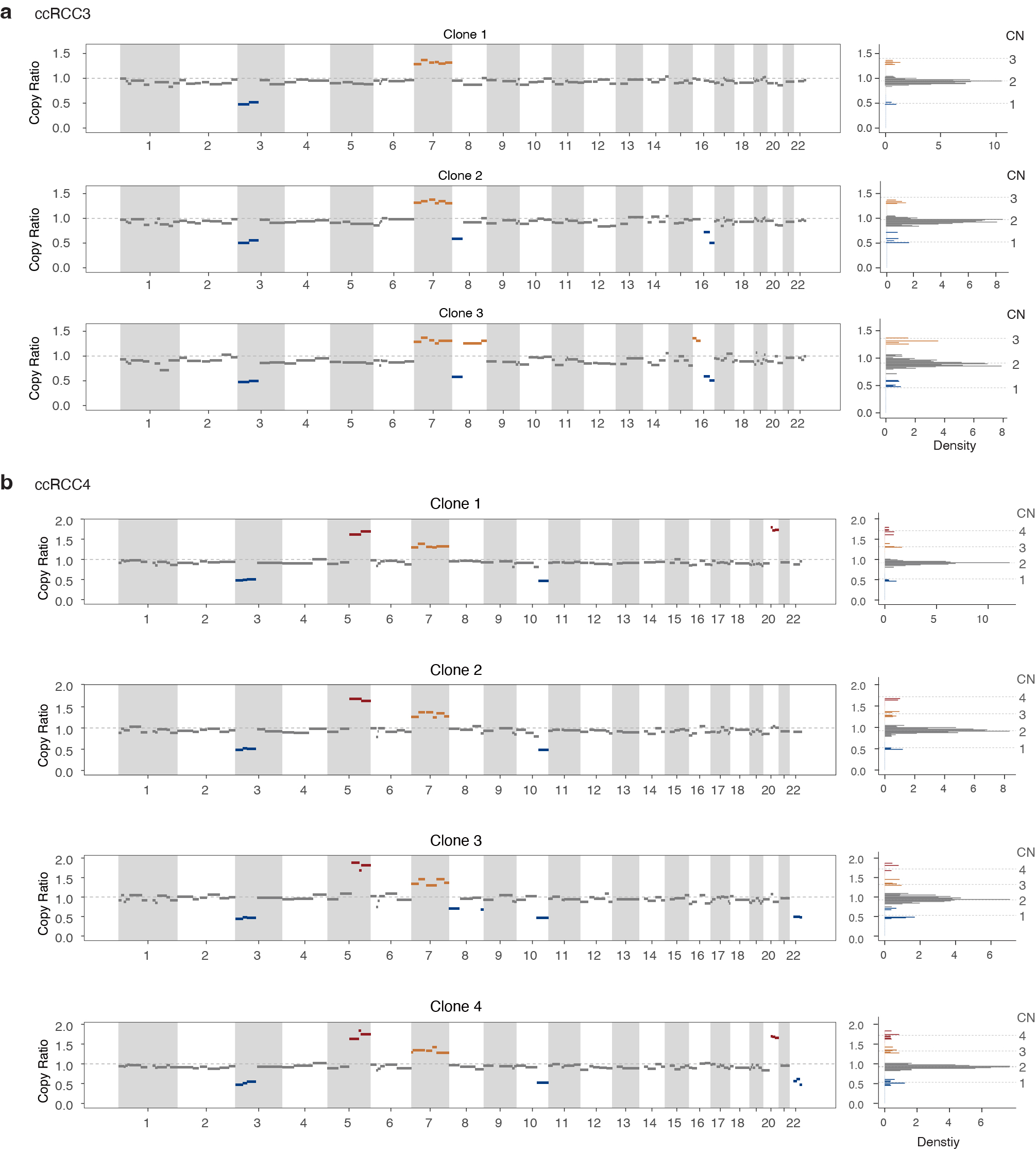


**Supplementary Figure 11.** The chromosomal segmental estimation for ccRCC3 (**a**) and ccRCC4 (**b**). Relative copy number profiles of each subclone (left) and the corresponding distribution of absolute copy number states (right).

**ALT TEXT:** Plots of chromosomal segmental copy-number estimation for ccRCC3 and ccRCC4, showing relative copy-number profiles for each subclone alongside corresponding genome-wide distributions of absolute copy-number states.

**
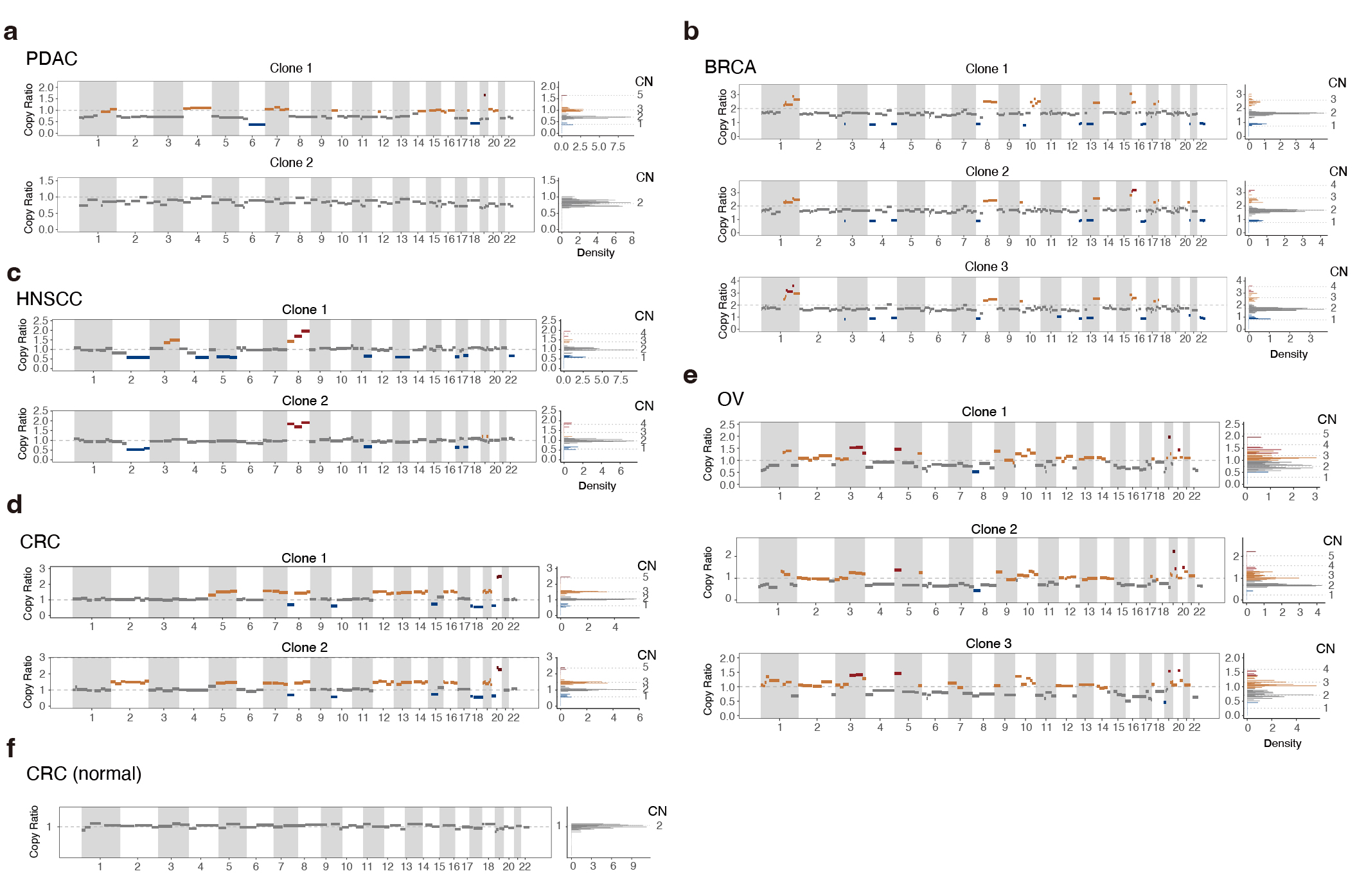
**

**Supplementary Figure 12.** TeaCNV-inferred chromosomal segmental profiles. (**a-e**) Results of PDAC, BRCA, HNSCC, CRC and OV samples. Each panel shows subclonal relative copy number profiles (left) and the distribution of absolute copy number states (right). (**f**) TeaCNV estimation for a normal CRC sample.

**ALT TEXT:** Chromosomal segmental copy-number estimation plots for PDAC, BRCA, HNSCC, CRC and OV samples, displaying relative copy-number profiles for each subclone together with corresponding genome-wide distributions of absolute copy-number states.


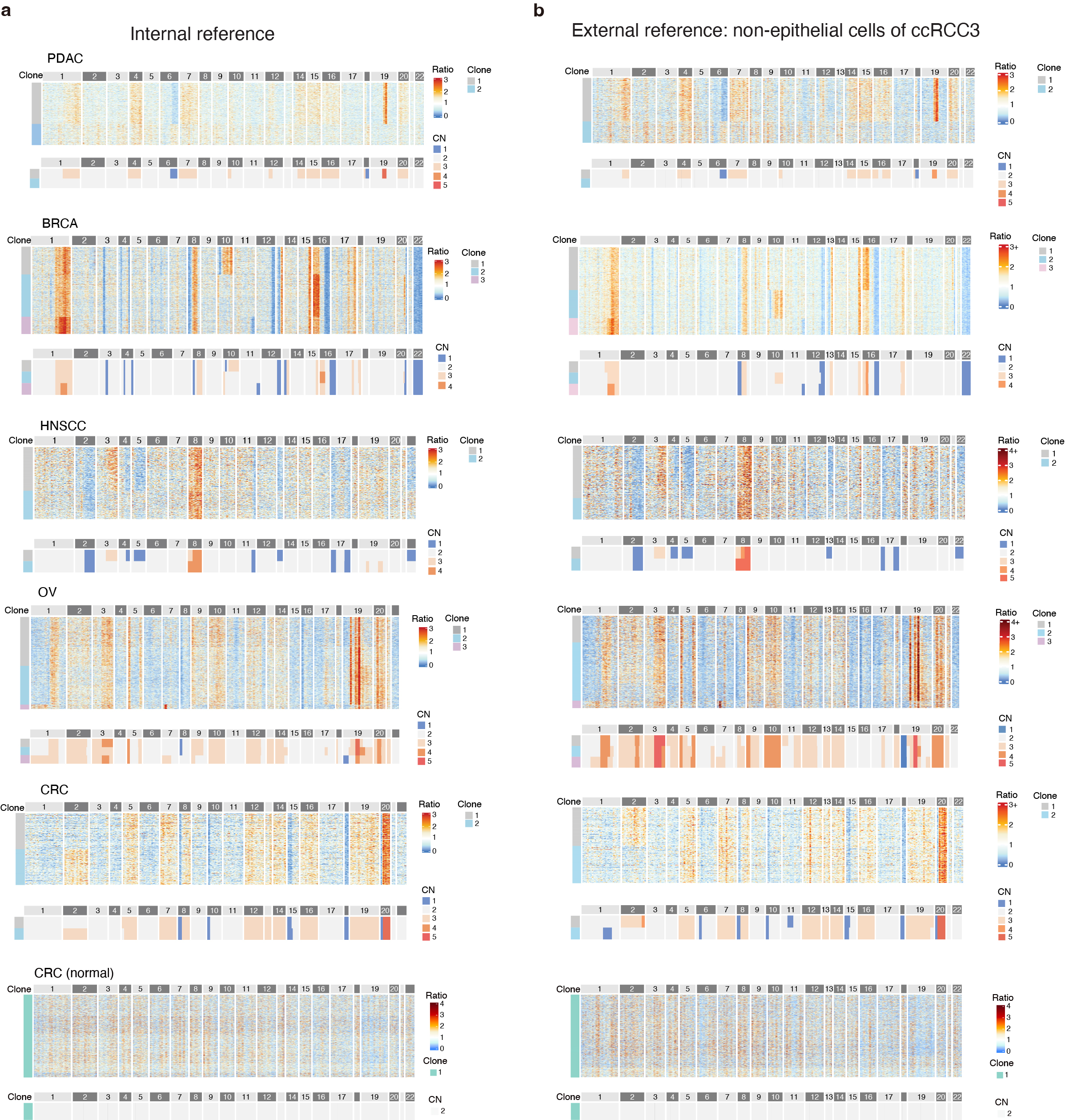


**Supplementary Figure 13.** CNV estimation by TeaCNV for multiple solid tumors using ccRCC3 as the reference. (**a**) TeaCNV-inferred copy number profile based on internal referencing for PDAC, BRCA, HNSCC, OV, CRC tumor and normal samples. (**b**) TeaCNV-inferred copy number profiles based on cross-dataset referencing, using ccRCC3-derived non-epithelial cells as an external reference for the corresponding samples.

**ALT TEXT:** A series of heatmaps showing CNV profiles for multiple solid tumors, including PDAC, BRCA, HNSCC, OV, and CRC. Panel (**a**) displays CNV profiles inferred using internal reference cells within each sample. Panel (**b**) shows the corresponding CNV profiles for the same samples, but inferred using external, ccRCC3-derived non-epithelial cells as a cross-dataset reference. Both panels display consistent chromosomal gain and loss patterns and similar subclonal architectures across all tested tumor types, including a diploid profile for the normal CRC sample.


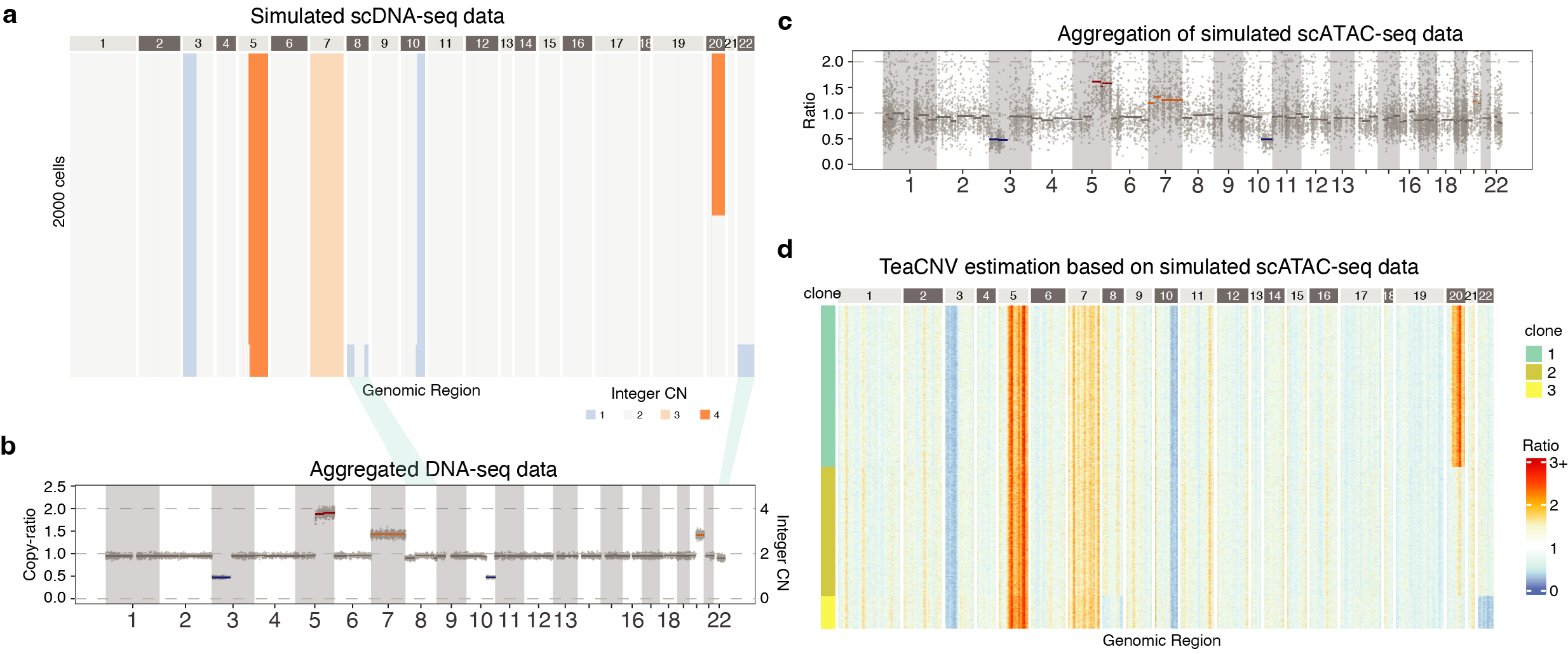


**Supplementary Figure 14.** Investigation of rare clonal CNVs. (**a**) Integer copy number (CN) profiles of simulated scDNA-seq data. (**b**) Relative and integer CN profiles of aggregated simulated scDNA-seq data. (**c**) Relative copy ratio profiles inferred from aggregated, paired simulated scATAC-seq data. (**d**) Relative copy ratio profiles and clonal substructure inferred by TeaCNV for the simulated scATAC-seq data.

**ALT TEXT:** Copy-number and copy-ratio plots from simulated datasets illustrating rare clonal CNVs, showing integer and relative CN profiles from simulated scDNA-seq (single-cell and aggregated), relative copy ratios from paired simulated scATAC-seq, and TeaCNV inferred copy-ratio profiles with corresponding clonal substructure.


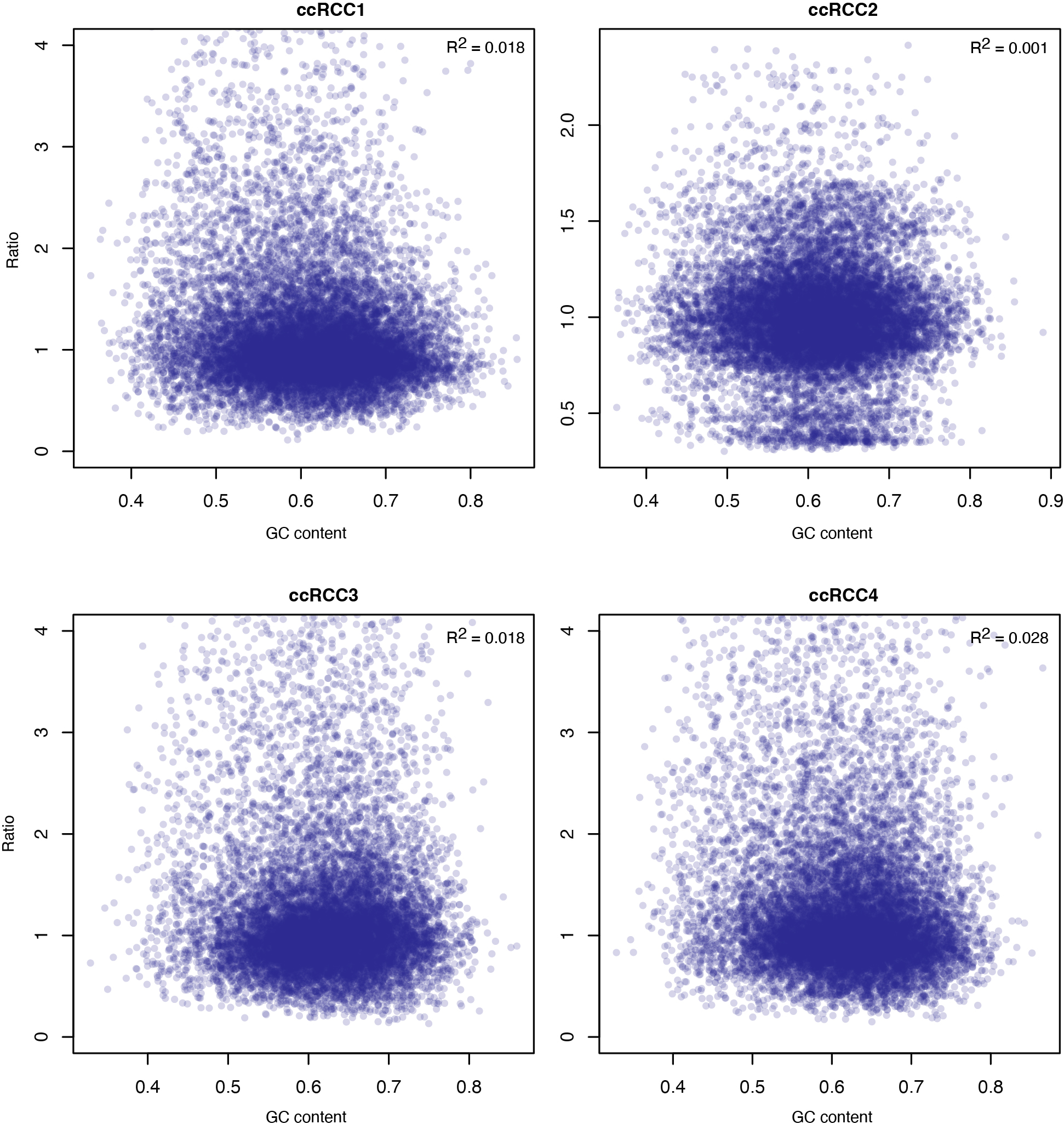


**Supplementary Figure 15.** The relationship between GC content and relative CNV signals (ratio). The R^2^ values were estimated using a linear regression model.

**ALT TEXT:** Scatter plots showing the relationship between GC content and relative CNV ratio signals, with fitted linear regression R^2^ values quantifying the strength of association.


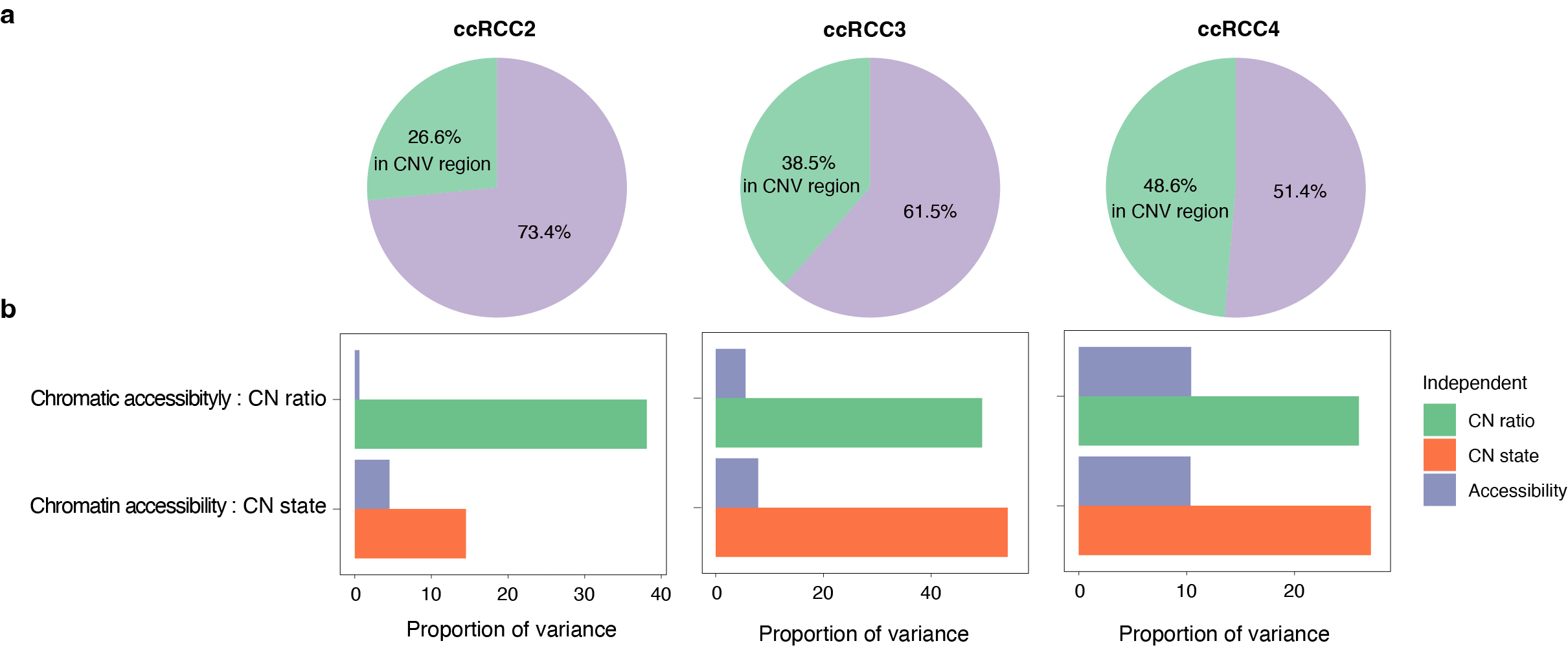


**Supplementary Figure 16.** Dissection of interplay between differential peaks (dePeaks) and CNVs. (**a**) The proportion of dePeaks located within or outside CNV regions for each ccRCC sample. (**b**) The proportion of variance in dePeak or CNV signals (CN ratio or CN state) explained by each other. Each bar corresponds to the proportion of variance in the factor labeled on the y-axis that is explained by the other factor as the independent variable.

**ALT TEXT:** Graphs dissecting the relationship between differential accessibility peaks and CNVs in ccRCC samples, showing the proportion of dePeaks within versus outside CNV regions and the fraction of variance in dePeak and CNV signals explained by each other.


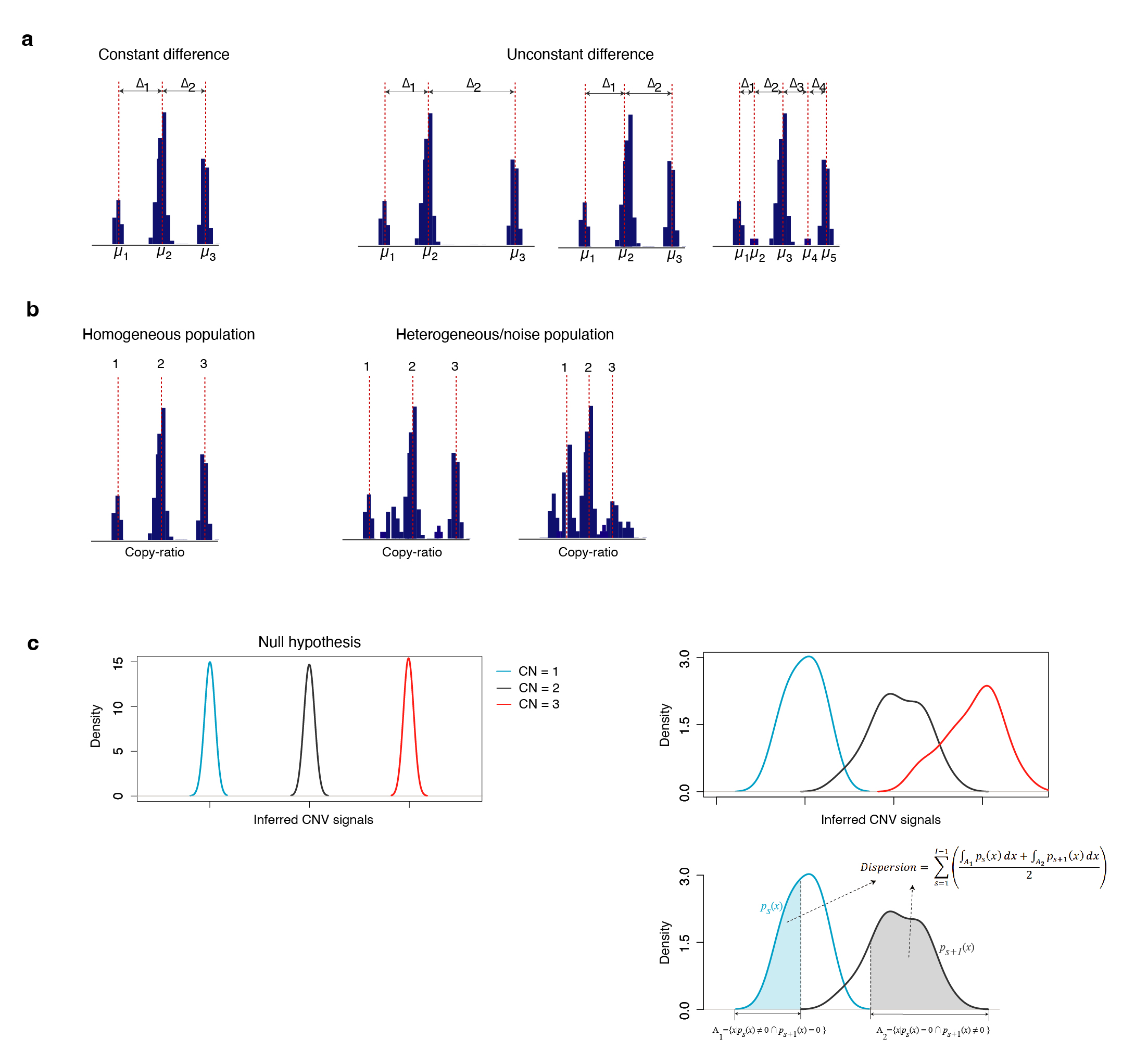


**Supplementary Figure 17.** Schematic illustration of optimizing absolute CN estimation. (**a**) Examples of inferred CN signals with constant (Δ₁ = Δ₂) or non-constant (Δ₁ ≠ Δ₂, …) differences between mean values ($\text{μ}_{\text{1}}$, $\text{μ}_{\text{2}}$, …), illustrating potential bias in the expected relative copy ratio for state $\text{q}$ ($\text{μ}_{\text{q}}$)estimation. (**b**) Relative copy ratio distributions from a homogeneous subclone (tight clustering at integer CN states) and heterogeneous/noise populations (broader, overlapping signals). (**c**) Dispersion scoring of CNV signals. Under the null hypothesis, CN states (CN = 1, 2, 3) form non-overlapping distributions (left). Noise leads to partial overlap (top right). The dispersion score quantifies separation between adjacent states (s, s+1) as the sum of non-overlapping probability density areas, normalized to [0,1] (bottom right). Higher scores indicate greater confidence in CN estimation.

**ALT TEXT:** Conceptual diagrams illustrating optimization of absolute copy-number estimation, including examples of CN signals with constant or varying differences between mean levels, contrasting relative copy-ratio distributions from homogeneous versus noisy or heterogeneous subclones, and a schematic of dispersion scoring that quantifies separation between adjacent CN states based on overlap of their probability density distributions.

**Supplementary Table S1.** Summary of characteristics for the simulated scATAC-seq datasets.

**Supplementary Table S2.** Performance metrics for CNV detection across simulated datasets.
